# Recurrent dynamics in the cerebral cortex: Integration of sensory evidence with stored knowledge

**DOI:** 10.1073/pnas.2101043118

**Published:** 2021-08-06

**Authors:** Wolf Singer

**Affiliations:** ^a^Ernst Strüngmann Institute for Neuroscience in Cooperation with Max Planck Society, Frankfurt am Main 60438, Germany;; ^b^Max Planck Institute for Brain Research, Frankfurt am Main 60438, Germany;; ^c^Frankfurt Institute for Advanced Studies, Frankfurt am Main 60438, Germany

**Keywords:** recurrent networks, neuronal dynamics, predictive coding, rate codes, temporal codes

## Abstract

Current concepts of sensory processing in the cerebral cortex emphasize serial extraction and recombination of features in hierarchically structured feed-forward networks in order to capture the relations among the components of perceptual objects. These concepts are implemented in convolutional deep learning networks and have been validated by the astounding similarities between the functional properties of artificial systems and their natural counterparts. However, cortical architectures also display an abundance of recurrent coupling within and between the layers of the processing hierarchy. This massive recurrence gives rise to highly complex dynamics whose putative function is poorly understood. Here a concept is proposed that assigns specific functions to the dynamics of cortical networks and combines, in a unifying approach, the respective advantages of recurrent and feed-forward processing. It is proposed that the priors about regularities of the world are stored in the weight distributions of feed-forward and recurrent connections and that the high-dimensional, dynamic space provided by recurrent interactions is exploited for computations. These comprise the ultrafast matching of sensory evidence with the priors covertly represented in the correlation structure of spontaneous activity and the context-dependent grouping of feature constellations characterizing natural objects. The concept posits that information is encoded not only in the discharge frequency of neurons but also in the precise timing relations among the discharges. Results of experiments designed to test the predictions derived from this concept support the hypothesis that cerebral cortex exploits the high-dimensional recurrent dynamics for computations serving predictive coding.

## Significance

This review attempts to unite three hitherto rather unconnected concepts of basic functions of the cerebral cortex, taking the visual system as an example: 1) feed-forward processing in multilayer hierarchies (labeled line coding), 2) dynamic association of features (assembly coding), and 3) matching of sensory evidence with stored priors (predictive coding). The latter two functions are supposed to rely on the high-dimensional dynamics of delay-coupled recurrent networks. Discharge rates of neurons (rate code) and temporal relations among discharges (temporal code) are identified as conveying complementary information. Thus, the new concept accounts for the coexistence of feed-forward and recurrent processing, accommodates both rate and temporal codes, and assigns crucial functions to the complex dynamics emerging from recurrent interactions.

Humans shift their gaze, on average, three times a second to take snapshots of their environment in order to collect the evidence required for a seamless reconstruction of the embedding visual world. This implies that the visual system is capable of accomplishing the following operations within fractions of a second: 1) extract from the two-dimensional and continuous distribution of electromagnetic waves on the retina the features that have proven useful during evolution for the analysis of visual scenes; 2) examine the relations among these features and compare them with stored information about meaningful constellations; 3) group features whose constellations are identified as meaningful—a function addressed as feature binding; 4) segregate such bound entities from each other and from the embedding background—a function addressed as scene segmentation or perceptual grouping; 5) identify the grouped feature constellations by matching them with stored knowledge about perceptual objects; 6) analyze the spatial and/or semantic relations among the recognized objects; and 7) store the content of these snapshots for later combination with previous and future snapshots in order to generate the percept of a coherent visual scene.

All of these functions require comparison of incoming sensory signals with stored information about regularities of the visual world—a function addressed as “unconscious inference” by von Helmholtz (1) and as predictive coding in the recent literature (2–7). This notion raises several challenging questions. Where and how is the internal model of the visual world stored? How is it possible to retrieve, within a fraction of a second, the specific priors required for the interpretation of a particular snapshot and to compare the ever-changing sensory evidence with stored knowledge? How is the factually infinite space of possible relations among features explored in order to segregate relevant from irrelevant relations? How are meaningful relations encoded and made amenable to further processing?

In the following sections, a concept will be presented that could account for some of these functions. It combines two complementary processing strategies: first, the analysis of relations in hierarchically structured feed-forward architectures and, second, the dynamic and context-dependent encoding of relations in recurrent networks. Subsequently, experimental evidence related to this concept will be reviewed. The data are taken mainly from anatomical and electrophysiological investigations of the mammalian visual cortex and include results of recent experiments designed to test the predictions derived from the proposed concept.

## Two Complementary Strategies for the Analysis and Encoding of Relations

The virtually infinite variety of perceptual objects results from variable combinations of a relatively small set of elementary features, just like the 26 letters of the Latin alphabet suffice to compose western literature. Therefore, cognitive systems need effective strategies to identify these features and to encode the relations among them.

### Encoding of Relations in Feed-Forward Networks.

One common strategy for the encoding of relations is based on the generation of conjunction-specific neurons in hierarchically structured feed-forward networks. Neurons tuned to elementary features distribute their responses through divergent and convergent connections to neurons of the respective next layer. By appropriate recombination of these connections and adjustment of their gain, the nodes of higher layers become tuned to represent specific feature constellations. In the mammalian visual cortex, for example, orientation-selective edge detectors receive convergent input from retinal ganglion cells with colinearly aligned receptive fields (RFs) (8–10), and thereby capture the prevalence of elongated contours in natural environments (11). In the mammalian visual cortex, this strategy is iterated over numerous processing stages (12). By varying the degree of overlap and the gain of the connections, neurons ultimately become selectively responsive to the highly complex constellations of features characterizing natural objects (13–17). Because these conjunction-specific nodes always signal the same constellations of features, this strategy of analyzing and representing relations is addressed as the *labeled line code*.

At low levels of the visual processing hierarchy, axonal convergence is mainly determined by genetic programs and leads to Gabor-like RFs. These capture elementary features of natural scenes and accord with the coding principles of independence and sparsity (18, 19). However, during early postnatal development, the feed-forward connections are highly susceptible to experience-dependent selection following a Hebbian modification rule (20, 21). Axons conveying correlated activity stabilize selectively on common target nodes. Due to this essentially self-supervised pruning process, nodes become tuned to respond preferentially to frequently occurring constellations of features (20, 22–24). Consequently, the responses of conjunction-specific neurons capture the statistical regularities of the visual environment (25). Some of these adaptive processes are carried over into adulthood and support perceptual learning (26). At lower levels, these learning processes serve the fine-tuning of feature selectivity (27–29) and, at higher levels, the generation of nodes responding to complex constellations of features (30). This strategy for the encoding of relations is also the hallmark of artificial neuronal networks, designed for the recognition and classification of patterns (31–33). The recent and highly successful developments in machine learning known as “deep learning convolutional networks” (34–36) capitalize on the scaling of this principle in large multilayer architectures and on the additional implementation of supervised learning algorithms (37).

However, labeled line coding has limitations. One is the high hardware cost. To achieve invariance, to cope with the nested relations characterizing natural scenes, and to encode context, an astronomical number of conjunction-specific nodes would be required—a problem addressed as combinatorial explosion. Another shortcoming of feed-forward networks is their lack of a temporal dimension. This makes it difficult for them to cope with temporal sequences and to establish relations among temporally noncontiguous events. To cope with this problem, modules have been added that have memory functions such as long short-term memory (35, 38, 39).

### Dynamic Encoding of Relations in Recurrent Networks.

A complementary strategy to capture relations among components relies on dynamic combinatorial codes, similar to those used by natural languages. This strategy was proposed by Donald Hebb (40) more than half a century ago. It posits that relations among components should be encoded by transiently binding feature-selective neurons into functionally coherent assemblies, the Hebbian assembly. Through cooperative interactions, these neurons would collectively signal the presence of a particular constellation of components. The formation of such transient assemblies requires self-organized cooperativity among network nodes and is therefore difficult to implement in feed-forward architectures. By contrast, the required interactions can be realized elegantly in recurrent network architectures. In theory, recurrent networks can be rolled out and simulated by feed-forward networks. However, this is only possible if the reciprocal interactions can be discretized, which may not be the case in natural systems. But, even then, a very large number of layers would be needed to represent the results of the recursive updating steps. For natural systems, this is not an option, because of the immense hardware costs and the constraints on processing speed.

Recurrent architectures are ubiquitous in nervous systems and particularly evolved in structures of vertebrate brains like the cerebral cortex (41, 42). According to Hebb´s proposal, the recurrent, reciprocal connections must be endowed with correlation-sensitive synaptic plasticity mechanisms (Hebbian synapses) in order to stabilize, preferentially, assemblies representing frequent constellations of features. With exposure to natural visual scenes, nodes tuned to frequently cooccurring features would become coupled more strongly and therefore engage more readily in cooperative interactions when activated by the respective feature constellation. In this coding scheme, conjunctions of features are represented by groups of temporarily cooperating nodes rather than by individual conjunction-specific neurons.

The ensemble coding strategy has a number of advantages that ideally complement those of feed-forward processing. One is the combinatorial nature of the code which economizes on hardware. Nodes can be flexibly and dynamically recombined to capture different constellations of features. Moreover, this strategy expands the storage space for the internal model because information about the statistical contingencies of features in natural scenes is now encoded not only in the topology and synaptic weights of feed-forward connections but also in those of the recurrent connections. Quantitative data on the cortical connectome indicate that these recurrent connections outnumber, by far, the feed-forward connections (41, 42).

Another advantage of assembly coding is that it can exploit not only discharge rate but also spike timing to convey information. This expands coding space and accelerates processing speed. Donald Hebb (40) had initially proposed that nodes forming a cooperating assembly should be distinguished by joint increases of discharge (firing) rate. And there is evidence that recurrent interactions can enhance discharge frequency (43). However, the notion that cooperating neurons should be distinguished solely by joint increases in discharge rate has been challenged (for a review, see ref. 44). First, different simultaneously active and spatially intermingled assemblies are difficult to distinguish from one another if all neurons participating in assemblies simply discharge more vigorously—a complication addressed as the “superposition problem” (45, 46−47). Second, increases in discharge rate are an ambiguous signature for a relational code because discharge rates also reflect stimulus energy and/or matches between stimulus and RF properties. Third, distinguishing cooperating neurons on the mere basis of enhanced discharge rates slows down processing speed because discharge rates of cortical neurons are low and can carry only a little information when integrated over short intervals. The latter problem could, in principle, be solved by configuring nodes as clusters of cells with similar feature selectivity and by averaging across their activity. However, this strategy is costly in terms of hardware and energy. In addition, it is hampered by the fact that fluctuations of cortical activity are correlated (“noise correlation”). Thus, averaging amplifies not only the signal but also the noise (43, 48).

## The Binding by Synchrony Hypothesis

In the 1980s, a serendipitous observation in the visual cortex of awake kittens led to a discovery that offered solutions to the problems associated with rate-coded assemblies. Neurons in the visual cortex activated by continuous contours were found to synchronize their spike discharges with millisecond precision even if located in functional columns segregated by up to 7 mm (49, 50). Because synchronization enhances the impact of discharges in downstream targets (51–53), it was proposed that the joint increase in salience of precisely synchronized discharges rather than joint rate increases would identify the transiently cooperating nodes of an assembly. This temporal code would substantially reduce the superposition problem because coincidence-sensitive downstream neurons can distinguish between different synchronous events with high temporal resolution (refs. 44, 46, 47, and 54; for reviews, see ref. 55). For the same reason, encoding relations by coincident firing rather than joint rate increases would allow for much faster detection of cooperating nodes (56) and also render the signature of relatedness independent of rate fluctuations.

### Synchrony and Oscillations.

Early studies on spike synchronization in the visual cortex used very simple stimuli such as moving light bars and gratings. For reasons discussed later, these stimuli, in particular the gratings, not only induce synchronous firing but also cause an oscillatory modulation of population activity in the gamma frequency range. Because increases in oscillatory power and spike synchronization covary in these special conditions, the important distinction between oscillatory patterning and spike synchronization got blurred, and the two phenomena were often regarded as equivalent (57). Oscillations are particularly easy to detect in signals that reflect the summed activity of large neuron populations such as are obtained with field potential, electrocorticogram, EEG, and MEG recordings. Although these signals reflect mainly synaptic currents, the power of narrow-band oscillations and the coherence of oscillations recorded from different sites are commonly considered as measures of spike synchronization. Consequently, band-passed oscillations and their coherence rather than the timing relations among discharges of distributed neurons became the target variable in numerous studies investigating response synchronization (for review of the extensive literature on oscillations, see refs. 58–62). Yet, what matters for information processing are not the oscillations but the rate and the precise timing relations of discharges. As detailed below, oscillatory mechanisms do play an important role in the temporal patterning and coordination of discharges (63–68), but oscillations, per se, likely convey no information.

### The Relation between Gestalt Rules and Response Synchronization.

Analysis of the synchronization phenomena observed in the visual cortex revealed that neurons tuned to features that have a high probability to cooccur in natural environments transiently synchronize their discharges with millisecond precision when coactivated by such related features. This was the case for continuous contours, colinearly aligned contour segments, contours sharing the same orientation, and contours moving with the same speed in the same direction (55). These feature constellations correspond to the basic Gestalt criteria for perceptual grouping, suggesting that synchronization probability reflects the statistical regularities of natural environments and serves feature binding (for a review, see ref. 44). Interestingly, this grouping by synchronization turned out to be dynamic and context sensitive: Typically, when a single elongated moving contour is presented, all nodes activated by this contour synchronize their discharges (50, 69). However, when two contours with different orientations overlap in space and move in different directions, the activated neurons split up into two groups (70–72). The neurons within each group discharge in synchrony, but there is no correlation at millisecond time scales among the discharges of neurons belonging to different groups. This self-organized grouping depends on the preferences of the neurons for the two contours. Those neurons join the same synchronization group that shares preferences for the same contour. Thus, the synchronized firing of neurons is context sensitive and signals whether the neurons are activated by a single “object” or two different “objects.” As discharge rates of all neurons were elevated to the same extent, this segregation of neurons into two distinct groups would not have been detectable by decoding only rate responses. Simulations of recurrent networks with enhanced coupling of nodes tuned to groupable features have reproduced such context-dependent synchronization phenomena (73–75). The results of these and related studies eventually led to the proposal that transient and context-sensitive synchronization of discharges could serve as a mechanism for perceptual grouping and inspired the “binding by synchrony” (BBS) hypothesis (76).

### Counterarguments.

In subsequent years, the BBS hypothesis has been challenged (77 and 78). As the early findings suggested a close association between oscillations and synchrony (see above), most arguments focused on the coherence of sustained oscillations rather than on the synchrony of discharges. Because oscillations are only sustained and stable in frequency under special stimulation conditions (79), vary in frequency as a function of stimulus energy and visual field eccentricity (77), and, if synchronized globally, limit the capacity of information transfer, oscillation-based synchrony was considered an epiphenomenon and irrelevant for information processing (for a review, see ref. 80). However, more-recent investigations in awake animals have revealed that synchronization of discharges, while often associated with oscillations, is not dependent on the occurrence of sustained oscillatory activity. Typically, episodes of synchronized firing are transient, occur in short bouts, follow at irregular intervals, and, if associated with oscillations, the oscillations tend to persist only over a few cycles (81–84). Similar results have been obtained in simulations of recurrent networks. Depending on the dynamic state of the network, synchronous events can occur in conjunction with brief bouts of oscillatory episodes but sometimes also in the absence of detectable oscillations (85, 86). Further evidence for the fast emergence and dissolution of stimulus-specific correlation structures in population responses of cortical neurons is discussed below when reviewing the effect of stimuli on cortical dynamics in awake monkeys.

Another argument against the BBS hypothesis was derived from the finding that, in area V1, synchronization did not comprise the entire population of spatially distributed neurons activated by a perceptual object (87). This argument assumes that the formation of object-specific Hebbian assemblies is achieved already at low levels of the visual processing stream. However, since neurons in the primary visual cortex represent only very elementary features, the grouping operations performed at this level can achieve only the low-level binding functions required for scene segmentation and perceptual grouping. Evidence actually suggests that correlation-based binding of attributes of perceptual objects does occur, but only at higher levels of the ventral visual processing stream, where individual neurons are already selective for object-specific feature constellations (88).

### The Anatomical Substrate of Synchronization.

The anatomical substrate for the transient, feature-specific synchronization of neurons in the primary visual cortex is, in all likelihood, the network of intracortical recurrent connections that extend tangentially to the laminae, are particularly abundant in supragranular layers, and link, preferentially, nodes responding to features which frequently cooccur in natural scenes (86, 89–96). Direct evidence for the synchronizing effect of these reciprocal cortico–cortical connections has been obtained by severing callosal connections. These connections share numerous similarities with the tangential intraareal connections (94, 97, 98) and likely serve the same functions, albeit across the two visual hemifields. Severing the callosal connections disrupts feature-specific synchronization between neurons that are located in different hemispheres and respond to contours crossing the vertical meridian (99). Another noteworthy result of this experiment was that interhemispheric synchronization of spike discharges had occurred with zero phase lag despite the considerable conduction delays imposed by the callosal fibers. This proves that reciprocal interactions can support zero phase lag synchronization despite coupling delays, a phenomenon that has also been confirmed by simulation studies (100–104) and is of great relevance for the encoding of relations by coincident firing.

Together, these observations suggest that the cooperative and virtually simultaneous interactions among neurons of recurrent networks can be exploited to encode relations among features, using temporal coordination of spiking activity (spike timing) as a tag of relatedness—a tag that permits faster and less ambivalent encoding and readout of relations than joint rate increases.

## Rate and Temporal Codes Convey Complementary Information

### Combination of Feed-Forward and Recurrent Processing.

**Fig. 1.**
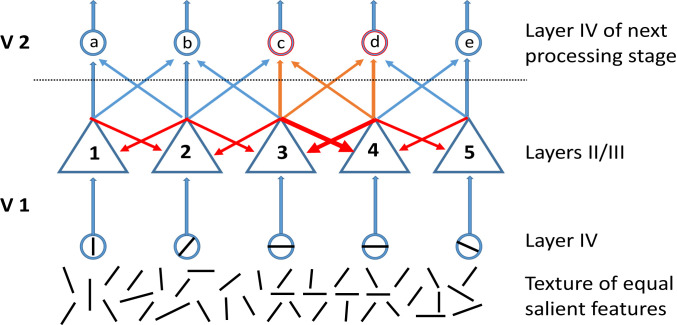
A cluttered scene, consisting of oriented contours (bottom), leads to activation of orientation-selective cells (blue circles) in input layer IV of V1. Because all contours have the same contrast and shape, the layer IV cells with corresponding RFs are assumed to be activated equally and to drive pyramidal target cells (1–5) in layers II/III via ascending feed-forward connections (blue arrows). These pyramidal cells are reciprocally coupled through excitatory collaterals (red arrows) of axons (blue and orange arrows) projecting to layer IV target cells (a–e) of the next processing stage (V2). Pyramidal cells 3 and 4 are coupled more strongly than their neighbors because they have a shared preference for colinearly aligned contours of the same orientation (indicated by thicker recurrent collaterals). This enhanced coupling is due to prior exposure to elongated contrast borders that are frequent in natural environments (for details, see *The Binding by Synchrony Hypothesis*). Because of strong coupling, cooperativity between neurons 3 and 4 is enhanced, and their responses become more coherent (indicated by orange color of feed-forward axons). This, in turn, leads to enhanced responses in target cells c and d, because cortical cells are driven more effectively by synchronous than temporally dispersed excitatory postsynaptic potentials (EPSPs) (51–53, 228). In addition, the discharges of neurons c and d are likely to be more synchronized, as well. In this way, recurrent coupling that captures the statistical regularities of natural environments can be exploited to detect relations among features, to convert these relations into temporal relations among discharges that, in turn, impact the spike responses of input cells at the next processing stage.

The coexistence of feed-forward and recurrent processing architectures in the brain raises the question of how these two processing strategies are combined with one another. The scheme depicted in Fig. 1 illustrates a possible scenario that exploits the respective advantages of the two processing strategies.

The example depicted in Fig. 1 illustrates how prior “knowledge” about environmental regularities, stored in the coupling weights of the recurrent network, can support the grouping of features by modulating the temporal coherence of responses to thereby evoke different rate responses at the input stage of a downstream area. In this example, all stimuli are assumed to have the same salience, and all feed-forward connections are assumed to have the same gain. Consequently, all nodes of the recurrent network, whose feature selectivity corresponds to matching features in the scene, receive the same excitatory drive. However, the grouping operations accomplished by the recurrent network enhance the temporal coherence of responses to “related” stimuli, in this case, colinearly aligned contours—and this leads to increased firing of the coincidence-sensitive target cells in the downstream area that receive synchronized input.

This simple grouping operation can, of course, also be accomplished in a strictly feed-forward architecture. However, the hardware costs are substantial because all possible combinations of colinear contours would have to be represented by individual conjunction-specific neurons. This combinatorial explosion can be avoided by dynamic binding, because individual neurons can be associated flexibly to signal different feature constellations. Moreover, delegating grouping operations to the dynamic interactions among nodes provides the additional option to render local grouping operations dependent on global context. In recurrent networks, local perturbations propagate across the whole network, often giving rise to traveling waves and avalanches that mediate interactions over large distances (105–109). Hence, local grouping (synchronization) probabilities depend not only on the coupling strength of the directly connected nodes but also on the dynamic state of the whole network. As this state reflects the match between sensory evidence and the entirety of the weight distributions of the recurrent connections, the numerous relations among contours of cluttered scenes can be evaluated in parallel to disambiguate local grouping options. Experimental evidence demonstrating the power of this highly parallel computation of grouping probabilities is discussed below.

### Rate and Temporal Codes Convey Complementary Information.

The proposed combination of feed-forward and recurrent processing also assigns complementary roles to changes in discharge rate (rate code) and precise spike timing (temporal code), respectively. Firing rate and synchronization can vary independently (110–114), and hence the two variables can, in principle, be used in parallel to convey different information. Two recent studies in awake monkeys (111, 115) reported that stimuli whose features match predictions provided by context mainly enhance synchrony, whereas nonmatching stimuli strongly increase discharge rate. In these studies, the feature space was color rather than orientation. Monkeys were shown large monochromatic surfaces, and these induced strikingly strong, oscillatory synchronization among the discharges of all simultaneously recorded, responsive neurons in visual area V1. In contrast, increases in discharge rate were moderate. Here the feature, in this case, the color, present in the RF centers of the responding neurons was fully predicted by the embedding context. If, however, the stimulus in the RF center was segregated from the surround by a gray ring or if the center stimulus had a different color, the respective neuron responded with a strong increase in discharge rate but no longer fired in synchrony with cells responding to the embedding background. In agreement with the proposal of Vinck and Bosman (116), this indicates that nodes activated by stimuli that match the ”predictions” provided by context engage in cooperative interactions and identify themselves through synchronization as coding for mutually predictive features. By contrast, cells activated by a nonpredicted feature signal this “prediction error” by a strong increase in discharge rate and do not synchronize with the neurons responding to the nonmatching surround. This agrees with the BBS hypothesis and, as depicted in Fig. 1, assigns complementary roles to increases of discharge rate and synchrony. The synchronously discharging neurons signal feature constellations that match priors while vigorously discharging neurons, whose spikes are uncorrelated signal prediction errors. Whether these complementary signals are relayed through different channels across downstream areas awaits further exploration.

So far, I have only discussed how recurrent networks can support dynamic grouping of features by establishing temporal correlations among distributed responses. However, the high-dimensional dynamics unfolding in such networks offer many more and fascinating options for computations. In the following paragraphs, I shall, therefore, discuss some of these computational capacities, derive predictions, and review evidence, partly from experiments designed to test these predictions.

## Computations Exploiting the Dynamics of Recurrent Networks

The complex dynamics generated by recurrent networks are exploited by a subclass of AI strategies addressed as “reservoir computing” whose instantiations have become known as “echo state” or “liquid” computing (117–122). Although these artificial networks lack many of the essential features of their biological counterparts, they have greatly inspired the exploration of computational capacities provided by recurrent processing in the cerebral cortex (123–128). The basic operation consists of transforming low-dimensional input patterns into high-dimensional dynamic states (dimensionality expansion) and using these high-dimensional patterns as input to classifiers. By this transformation, stimuli that overlap in low-dimensional space become linearly separable.

**Fig. 2.**
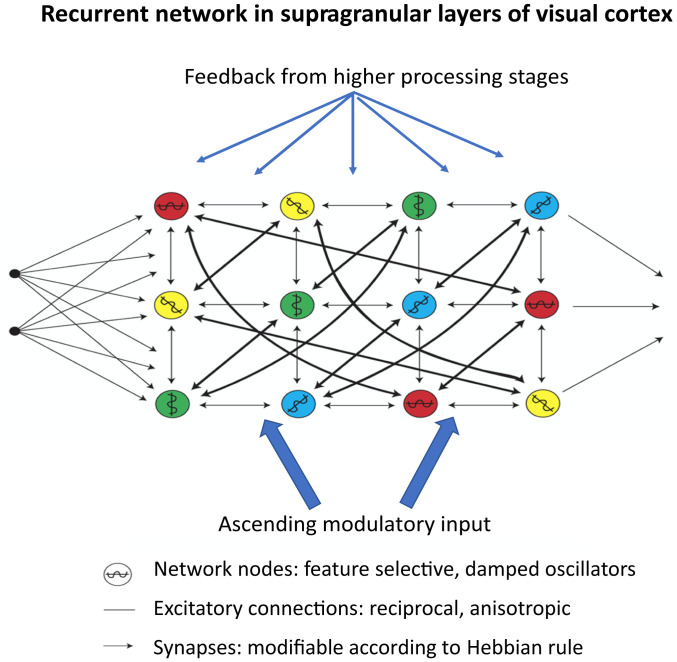
Simplified graph of recurrent connections among functional columns in layers II/III of the primary visual cortex. Nodes with similar colors respond to features that frequently occur together and are therefore coupled more strongly than other nodes (thick lines).

While sharing this essential capacity, natural recurrent networks such as are realized, for example, in the superficial layers (II and III) of the mammalian primary visual cortex differ in essential aspects from their artificial counterparts. These differences are summarized in Fig. 2. 1) The network nodes are feature selective, most of them showing shared and graded selectivity for different feature domains [“mixed selectivity” (129)]. This mixed selectivity probably arises from convergence of heterogeneous feed-forward connections that become stabilized selectively during development because they respond to frequently cooccurring features. Here, only the orientation of visual contours is considered. 2) The nodes have a propensity to oscillate because a fraction of cells have pacemaker properties (130, 131) and because local inhibitory feedback circuits (60, 61, 132) support rhythmic discharge patterns. 3) The topology and gain of the recurrent connections are anisotropic because these connections exhibit spatial gradients and are susceptible to experience-dependent modifications (see above). Hence the coupling of nodes reflects the probabilities with which the nodes have been coherently activated in the past and captures the statistical regularities of the visual world. And the mixed feature selectivity of the nodes further increases the dimensionality of the representational space (see above and ref. 133).

### The Challenge of Stability.

The dynamics of recurrent networks requires subtle control to assure stability and to adjust time constants to the actual processing needs. On the one hand, the network must be prevented from engaging in runaway dynamics and becoming epileptic. On the other hand, a critical level of spontaneous activity has to be maintained to accelerate the readout of stored information and the grouping of nodes. If the nodes were solely activated by sensory input, these processes would take time to self-organize or might not occur at all. Nature adjusts the dynamics of cortical networks with a number of self-regulating local mechanisms involving complex networks of inhibitory neurons (E/I balance) (134), normalization of synaptic strength (135), synaptic plasticity (136), adaptation (137), and membrane resonance (138). In addition, excitability is controlled by globally acting modulatory systems (139, 140). These control mechanisms are not only essential for stability but also allow task-dependent optimization of network dynamics for the execution of particular computations. Experimental evidence indicates that cortical networks operate in a dynamic regime close to criticality (107, 108, 141, 142). Varying the distance of the network dynamics from the critical bifurcation point toward chaotic behavior offers interesting options for computations at the edge of chaos (143). It allows control of 1) the networks “fading memory,” that is, the duration of the interval over which temporally segregated inputs can interact with one another; 2) the speed of state transitions, that is, the rate at which cooperative interactions among nodes can be established and dissolved; and 3) the distance over which nodes can interact with one another (144).

## Storage and Readout of Priors

Theories of perception, formulated more than a hundred years ago (1), and a plethora of more recent experimental evidence (145, 146) indicate that perceiving is a constructivist process. An internal model of the world is used to interpret sparse and noisy input signals, to make inferences about their relevance, and to eventually identify and classify perceptual objects. Given the vast number of priors required for the interpretation of ever-changing sensory input patterns, the store harboring this model must have an immense capacity. Moreover, the store must permit ultrafast retrieval of the relevant priors to meet the constraints of processing speed (see the Introduction).

In the following, a scenario is proposed that exploits the dynamics of recurrent networks for the storage and retrieval of priors, and for the ultrafast comparison of stored knowledge with sensory evidence.

### Informative Spontaneous Activity.

Spontaneous activity of the cerebral cortex is highly informative. Even in sensory areas, it reflects global states of the organism and is influenced by movements (147, 148). In addition, it exhibits a distinct correlation structure that reflects the motifs of recurrent connectivity, that is, the topology and the weight distributions of the connections among nodes (149–151). Hence, in V1, the correlation structure of spontaneous activity covertly represents priors about regularities of natural scenes (152). In addition, this correlation structure is expected to be shaped by recurrent connections from higher processing levels (153, 154) that contribute information about higher-order statistics of natural scenes (155). Hence, resting activity in V1 is supposed to be high dimensional and to occupy a vast but constrained subspace inside the universe of all theoretically possible dynamic states.

### Matching Sensory Evidence with Priors.

Once sensory signals become available, they are likely to trigger the following cascade of effects: They drive, in a graded way, the subset of nodes tuned to the features contained in the actual input pattern and thereby perturb the ongoing network dynamics. If these feature constellations match some of the priors stored in the anisotropic coupling connections, then a subnetwork of nodes will immediately resonate and engage in enhanced cooperation. Consequently, the network dynamics collapses to a specific substate. This substate is bound to have a lower dimensionality than the spontaneous activity because its dynamics is constrained by the enhanced cooperation of a subset of nodes. Due to reverberation, activity will quickly spread across the recurrent network, and stimulus-specific substates will eventually involve not only the initially driven nodes but, as time elapses, also other nodes of the network. Which of these additional nodes will be engaged depends, again, on the weight distributions of the recurrent connections and on the constellation of features in the stimulus. The evolution of a particular substate is therefore determined not only by the stimulus but by the entirety of the synaptic weights of the recurrent connections, that is, the priors stored in the functional architecture. The attained substate is thus equivalent to the result of a matching operation that combines the actual sensory evidence with a vast amount of stored “knowledge.” Therefore, it contains more information than the sensory evidence alone. Because these processes occur within a very high-dimensional state space, substates induced by different input patterns are likely to be well segregated and easy to classify once the respective substates have sufficiently diverged from resting state.

In the following paragraphs, predictions are derived from this scenario and related to experimental evidence.

## Predictions and Experimental Evidence

The evidence reviewed in the following section is drawn partly from the literature and partly from experiments designed specifically to test the above predictions.

### Prediction i.

Knowledge about the statistical regularities of natural visual scenes should be stored in the anatomical layout and the synaptic weights of both feed-forward and recurrent connections. The regularities captured by the feed-forward architecture should be expressed explicitly by the specific RF properties of the network nodes. By contrast, the regularities stored in the weight distributions of the recurrent connections should not contribute to the classical RF of the nodes but rather mediate selective cooperative interactions. These weight distributions should, in addition, be reflected in the correlation structure of spontaneous activity.

As documented by the innate feature selectivity of nodes (14, 156), the genetically determined layout of feed-forward connections does capture certain regularities of visual scenes. As developmental studies indicate, these inborn response properties of nodes are complemented and refined by experience-dependent pruning of feed-forward connections (for a review, see refs. 157 and 158). The same holds for the recurrent connections. Their layout and synaptic weights are also specified by experience (159) and reflect statistical regularities of natural environments (86, 92–97). Most of these developmental pruning processes are self-supervised (160, 161); for a review, see refs. 157 and 158. They require activated brain states (162), require scanning of and interaction with the environment (163), and fail to occur when the available sensory evidence is in conflict with inborn response properties of the system (164, 165). However, some of these network changes can be induced even under anesthesia when stimuli are presented repetitively (162, 166). Because the recurrent connections from higher to lower cortical areas share essential features with the intraareal connections, it is likely that they are also susceptible to experience-dependent modifications, but this possibility is much less explored.

Evidence also supports the conjecture that spontaneous activity is informative and reflects the priors residing in the network architecture. The resting-state dynamics of cortical networks are high dimensional (167), exhibit spatiotemporal patterns expected to occur in recurrent networks such as traveling waves and avalanches (106–109), and operate in a dynamic range close to criticality (108, 109, 168). In agreement with the anisotropic layout of the recurrent connections, resting activity exhibits a covariance structure that reflects the differences in coupling strength among nodes (95, 150, 169). And, as expected from the experience-dependent adjustment of coupling, this correlation structure is susceptible to experience-dependent modifications during early development (170), remains malleable throughout life by learning (171, 172), and recapitulates regularities of the environment (152). Thus, prediction i is already well supported by data.

### Prediction ii.

In response to structured visual stimuli, the initially unconstrained, high-dimensional internal dynamics of the recurrent network should collapse into metastable subregions of the state space. These substates should exhibit reduced dimensionality, and their dynamic signatures should be stimulus specific. Stimuli matching the priors should induce substates, whose correlation structure, consistency, and persistence should reflect the quality of the match between sensory evidence and stored priors. Substates induced by natural stimuli should carry more stimulus-specific information than substates induced by less matching stimuli. Accordingly, classifiers should perform better for substates induced by natural than unnatural stimuli.

Stimulation reduces the variance and dimensionality of the dynamics, as indicated by a decrease of the Fano factor and an increase in coherence (155, 167, 173). This is consistent with the collapse toward lower-dimensional substates. For low levels of the visual system, a particularly good match with stored priors is achieved with grating stimuli because they comply with a large number of Gestalt criteria (priors). These are continuity, colinearity, regularity of texture, symmetry and similarity in feature space (in this case in the orientation domain), and—if the grating drifts—common fate. With drifting gratings, each stimulus entering a neuron’s RF is unequivocally predicted by the context. Accordingly, these stimuli elicit particularly stable and coherent states that are characterized by sustained, highly synchronized responses that, in addition, oscillate in the gamma frequency range (see above and refs. 49 and 50). If more-complex constellations of features are presented, such as plaids or several contours drifting in different directions, the correlation structure of the substates becomes more complex and less sustained (71, 174). And, if stimuli are presented that lack any regularities, such as random dot textures, and therefore do not match any priors, the resulting population responses show only weak, if any detectable, coherence (113). Accordingly, natural stimuli induce substates whose correlation structure exhibits greater stimulus specificity than substates induced with manipulated stimuli whose statistical regularities of higher order had been removed (115, 155). Recent experiments complement these findings by showing that substates induced with natural scenes allowed for better classification of stimulus identity with linear decoders than substates induced with phase-scrambled versions of the same natural scenes. This improved classification was not due to trivial causes such as enhanced signal to noise ratios, because discharge rates and stimulus-induced reduction of response variability (Fano factor) were exactly the same for natural and scrambled images. However, natural stimuli evoked stronger gamma bursts than their scrambled versions. This suggests that good matches between evidence and priors lead to more succinct, more coherent substates than poor matches and agrees with the proposal of Vinck and Bosman (116) and the findings of Peter et al. (111) and Uran et al. (115). The likely reason is that matching stimuli activate, preferentially, subsets of nodes that are strongly coupled. As a consequence, these will engage in stronger cooperative interactions and, when driven sufficiently, in oscillatory resonance.

The prediction that priors stored in the network contribute to the stimulus specificity of substates is also supported by the finding that classifiers trained to identify stimuli often perform better on the delayed, reverberating part of the responses than on the initial transients that are dominated by direct sensory input (175). Principal component analysis (PCA) of the rate vectors showed that the segregation of the vectors induced by different stimuli can increase over a few hundred milliseconds even after stimulus offset (175). The latter finding was obtained in experiments performed under anesthesia and requires confirmation in awake preparations.

A dependence of cortical dynamics on the match between sensory evidence and stored priors has also been described for another phenomenon that is characteristic of recurrent networks: the sequential activation of nodes after perturbations. A recent study (176) showed that natural scene stimuli lead to sequential activation of neurons (nodes) in monkey V1 and V4. These sequences (songs) are stimulus specific; that is, information about the identity of the stimuli is contained not only in the rate vector but also in the rank order of the activated nodes. Hence, stimuli can be identified by decoders trained to sequence order rather than rate vectors. A dependence of rank order on the match between sensory evidence and stored priors is suggested by the finding that the temporal precision of the sequences and hence also their decodability is higher for natural stimuli than for manipulated stimuli that lack some of the higher-order regularities of natural scenes.

Together, these observations support prediction ii that stimuli matching stored priors induce a collapse of systems dynamics toward metastable, low-dimensional substates that are more informative when induced by stimuli that match stored priors.

### Prediction iii.

Reverberating activity should outlast the duration of stimuli. Hence, substates evoked by successively presented stimuli should overlap. This should allow decoding of sequence order and identity of the sequentially presented stimuli at the end of the stimulation sequence.

Experiments with anesthetized cats revealed that linear decoders could determine the identity of both the first and the second stimulus as well as the sequence order of the stimuli from activity vectors persisting after the end of the second stimulus (118).

### Prediction iv.

Because the synapses of recurrent connections are plastic, repeated exposure of the network to the same stimuli should engage unsupervised or self-supervised learning processes. These should favor the stabilization and segregation of stimulus-specific substates. Consequently, discriminability of substates should increase with the familiarity of stimuli. Moreover, particularly well-stabilized substates might then emerge even in the absence of stimulation and appear as stimulus-specific correlation patterns in resting state activity (replay).

After frequent exposure to the same set of stimuli, performance of classifiers trained to discriminate between stimuli improved, suggesting enhanced orthogonalization of stimulus-specific substates. Accordingly, PCA analysis of the activity vectors revealed increased segregation of representations in the high-dimensional space for stimuli that had been presented particularly frequently (175). Also in agreement with this prediction is the evidence that stimulus-specific response vectors evoked by frequently repeated stimuli are replayed in the primary visual cortex during resting state. Classifiers trained to reconstruct stimuli from spontaneously occurring response vectors achieved much better reconstructions for stimuli that had been presented frequently than for less familiar stimuli (175); see also ref. 177. These experiments were performed under anesthesia, making an involvement of supervised or reinforcement-based learning processes unlikely. This interpretation is supported by simulations of self-organizing recurrent networks. If the recurrent connections are endowed with Hebbian synapses, they reproduce this improved classification of familiar stimuli (121, 175, 178). Direct evidence for unsupervised modifications of recurrent connections by repeated stimulation is also available from the cat visual cortex of anesthetized cats (166). Repeated exposure to drifting gratings led to an expansion of those orientation domains that were responsive to the conditioning stimulus. These changes involved modifications at the level of the tangential recurrent connections, supporting further the notion that correlated activation of network nodes enhances their reciprocal coupling.

### Prediction v.

Because Hebbian modifications of synaptic connections require a high amount of cooperativity (179), network states exhibiting a high degree of coherence should be particularly well suited to support this kind of nonsupervised learning.

The modifications of orientation domains induced with drifting gratings in the cat visual cortex (see preceding paragraph) occurred only when stimuli induced well-synchronized responses, in this case, based on gamma oscillations. When stimuli failed to induce synchronized activity but just induced temporally dispersed discharges of comparable frequency, the orientation domains did not change. Rather, the responses of the neurons driven by the conditioning stimulus showed a long-lasting decrease in amplitude (166) as is characteristic of habituation or long-term depression.

### Prediction vi.

Top-down signals associated with attention, expectancy, and prediction should influence the network’s dynamic state and thereby facilitate convergence toward substates that serve best the respective behavioral goal. This should be reflected by changes in the dimensionality and correlation structure of network activity and the speed of convergence.

With respect to attention effects on network dynamics of V1, evidence is still sparse. There is consensus that spatial attention exerts only weak to moderate effects on rate responses and the power and frequency of synchronized oscillations in the primary visual cortex. A consistent finding is a decrease of correlations (140, 180–183). This suggests that attention shifts network dynamics toward a state that permits rapid and minimally biased transitions into stimulus-specific substates once sensory evidence becomes available. The majority of studies investigating attention effects on dynamics concentrated on changes of coherence between visual areas (184–188). These studies were inspired by the communication through coherence hypothesis (58) which postulates that synchronization between the oscillatory activity of sender and receiver facilitates communication. Attention has been shown to increase coherence of oscillatory activity between V1 and V4 whereby coherence measures were based on local field potentials (LFPs) which reflect mainly synaptic activity (see above). It has been concluded from these results that transmission of the attended signals is facilitated by increasing synchronization between V1 and V4 (184, 186). This interpretation has recently been challenged by the demonstration that increases of LFP-derived coherence measures do not necessarily imply enhanced effective communication (189). The reason is that the LFPs in the target area are, to a large extent, due to synaptic currents caused by the afferents from the sending area, and, therefore, changes of the sender´s dynamics can mimic changes in coherence measures.

By contrast, strong effects on cortical dynamics have been observed by the expectancy of having to react to a rewarding stimulus. Monkeys were shown a sequence of three temporally segregated stimuli consisting of identical drifting gratings and were trained to respond swiftly to a change in the grating´s orientation. The color of the fixation spot indicated to the monkey whether the second or the third stimulus was going to change. In this case, the responses to the cued grating were associated with a large increase (several hundredfold) in the power of the gamma oscillations and synchronized firing compared to the grating that promised no reward. Despite these drastic changes in network dynamics, the discharge frequencies of the responses to the cued and uncued stimuli were similar, confirming again that discharge rates and the correlation structure of network dynamics can change independently (110). Thus, focusing attention on a stimulus that required a swift response to obtain a reward changed the correlation structure of the dynamic state induced by this stimulus. Unlike with spatial attention, this change was particularly strong when the rewarded stimulus had to be selected from a sequence.

In conclusion, available evidence supports many of the core predictions derived from the hypothesis that, for its computations, the cerebral cortex exploits the high-dimensional state space provided by the dynamics of recurrent networks. In the sections above, only low-level functions were considered. However, the proposed computational strategy can be iterated at all levels of the processing hierarchy, because all cortical areas possess the required recurrent circuits. At early levels of processing, the weight distributions of the reciprocal connections capture only the elementary Gestalt rules of perception (190), and the resulting grouping operations can therefore support only low-level functions such as feature binding, scene segmentation, and figure–ground segregation. Grouping criteria based on higher-order statistics of natural scenes are, in all likelihood, stored in the functional architecture of downstream areas. However, these can, through feedback connections, contribute additional context for the disambiguation of grouping operations in the primary visual cortex. It is only at the highest levels of the processing hierarchy, where nodes respond to complex constellations of features due to iterative recombination of feed-forward connections, that assemblies of transiently bound nodes could represent entire perceptual objects, abstract categories, concepts, or action plans. However, apart from the evidence that recurrent connections also have a distinct topology (191) in higher cortical areas, little is known about the principles that govern the layout of these connections. In this context, it is noteworthy that comparison between artificial and natural systems has revealed that certain functional properties of nodes in higher cortical areas can only be accounted for if feed-forward processing is complemented by recurrent processing (192).

## Putative Functions of Oscillatory Mechanisms

As mentioned above, stimuli that simultaneously drive a sufficient number of strongly coupled nodes induce prominent, well-synchronized oscillating responses. The reason is that interactions among nodes with a propensity to oscillate give rise to resonance and entrainment in synchronized oscillatory activity (82, 100, 193, 194). The propensity of cortical networks to oscillate is due to multiple mechanisms that operate at different scales. They range from individual neurons with pacemaker characteristics (131, 195), over microcircuits with recurrent inhibition (141, 196–206), to large-scale recurrent interactions (106). Therefore, oscillations are a hallmark of cortical activity, which raises the question of whether they are merely an unavoidable epiphenomenon of circuits serving specific functions such as gain control, normalization, noise suppression, etc., or whether they serve specific functions (refs. 77 and 78; for a review of controversial interpretations, see ref. 80).

Oscillatory mechanisms have an undisputed functional role in pattern generator networks that serve the control of movements. These networks possess neurons with pacemaker currents that generate and stabilize rhythmic activity (refs. 207–209; for a review, see ref. 61). Evidence indicates that a substantial fraction of neurons in the primary visual cortex of cats and monkeys also have properties of pacemaker cells (131, 195). These oscillate in the same frequency range as the ING and PING circuit (210), which suggests that they might serve to enhance and stabilize oscillatory resonance.

Oscillatory resonance amplifies and facilitates interactions, stabilizes dynamic states, and generates precise temporal relations among discharge patterns (205, 211). Facilitation of interactions by resonance might be required to assure communication among distant, not directly connected nodes that can only interact via polysynaptic paths. Such long-distance interactions are likely needed to accomplish context-dependent scene segmentation in large visual areas with a high magnification factor such as the primary visual cortex of cats and monkeys. This could be the reason why pacemaker cells are implemented in monkey and cat V1, which has a high magnification factor, but seem to be missing in small cortical areas such as the rodent visual cortex and higher visual areas of the monkey (131).

Another process likely to benefit from oscillatory resonance is synaptic plasticity. Its induction is facilitated by precise temporal alignment of discharges, strong cooperativity, and repetition of the change-inducing activity patterns (see above). All of these prerequisites are fulfilled if networks are seeded with oscillatory mechanisms that allow for resonance. These mechanisms stabilize dynamic states and establish precise temporal relations among discharges. The finding that experience-dependent synaptic modifications are facilitated in the visual cortex by entrainment of the network in synchronized gamma oscillations supports this interpretation (166).

Oscillatory mechanisms could also be exploited to establish precise temporal relations between responses that either lack distinct temporal structure or are of different origin. This is likely the case for all self-generated activity patterns, for example, the replay of memories, but also for responses to stationary stimuli or responses from different sensory modalities. When these activities have to be subject to binding operations for the establishment of new associations, a common temporal structure has to be imposed on neuronal discharges by internal mechanisms. This is necessary to make discharges contiguous in time and to comply with the exquisite sensitivity of synaptic plasticity mechanisms to temporal correlations. Thus, oscillatory modulation of discharges and resonance could be exploited to achieve the temporal coordination required for associative learning.

Finally, the stabilizing effect of oscillatory resonance may also serve to maintain informative states for a sufficiently long period of time to permit 1) their disambiguation by top-down signals from higher cortical areas and 2) the integration of temporally segregated stimuli for the analysis and encoding of sequences.

However, the flip side of oscillatory resonance is that it takes time to evolve and to dissolve and, if too generalized, destroys information. Thus, global entrainment of neuronal populations in synchronous oscillations as it occurs, for example, with the highly redundant grating stimuli may actually prevent transmission of signals in downstream areas. The inhibitory neurons at the input layer IV of downstream areas receive highly convergent connections from the respective upstream areas. Therefore, their tuning is broad, and they respond strongly to globally synchronized input. Thus, globally synchronized input from an upstream area likely prevents further transmission by blocking responses of the excitatory target cells in the downstream area (212). This is one of the reasons why pyramidal cells in the primary visual cortex respond poorly to diffuse flashes of light which elicit globally synchronized but spatially unstructured input from the lateral geniculate. Thus, global synchronization could act as a filter that suppresses redundant information. Whether synchronization of discharges facilitates or prevents propagation of signals may therefore depend critically on the spatial structure of the synchronized activity. Traveling waves are particularly interesting in this context, as they endow synchronized oscillatory activity with not only temporal but also spatial structure (102, 106) and have recently been shown to influence behavioral choice in primates in a signal detection task (213). In contrast to classical population codes, traveling waves are not separable as the product of a spatial function and a temporal function. This mathematical difference makes it possible to convert temporal information into a spatial code and vice versa (see ref. 102).

When discussing putative functions of oscillations, it is also important to consider that the propensity of cortical networks to engage in oscillatory activity is regulated by ascending modulatory systems (214–217) and top-down control (140, 182, 184, 203). These mechanisms allow adjustments of oscillatory resonance and thereby control the temporal and the spatial scale of interactions among distributed responses.

## Conduction Delays

Coupling delays among nodes are variable because recurrent connections differ in diameter, length, and myelination, raising the question of whether variable conduction delays of axons are exploited for computation or are an unavoidable epiphenomenon of neuronal hardware that the system has to cope with.

Conduction delays, if exceeding a critical threshold, may jeopardize oscillatory resonance and synchronization, prevent effective summation of convergent inputs at conjunction-specific nodes, and pose a problem for the time-sensitive synaptic plasticity mechanisms. On the other hand, conduction delays between the nodes may have computational advantages. Recurrent systems with delay are infinite dimensional and thus offer an immensely large coding space (218). Moreover, as shown by Muresan and Savin (138), delays stabilize activity in recurrent networks and may thus be an essential ingredient of recurrent processing. Adjustment of conduction velocities can serve to render inputs from different sources contingent in time, to generate sequences, and to produce functionally relevant phase offsets. Several observations suggest that these possibilities are exploited by the brain. Through variations in axon diameter and myelination, it is assured that impulses in bifurcating callosal fibers arrive at exactly the same time in the widely distributed terminal arbors (219). Likewise, conduction times of connections in the cat visual system between area 17, the suprasylvian cortex (PLMS), and the superior colliculus are tuned in a way that assures that signals that are correlated between area 17 and PMLS arrive simultaneously in the tectum despite differing path length (220). In the auditory system, delay lines assure coincidence of binaural signals on common target cells in the mesencephalon to achieve direction tuning for auditory targets (221). These few examples indicate that neuronal connections are specific not only in the spatial but also in the temporal domain. How this specificity is achieved is little explored. One possibility is activity-dependent selection of afferents during development by the established correlation-sensitive synaptic plasticity mechanisms. As these stabilize, selectively, afferents whose activity is correlated in time, they can serve to select, among axons with different conduction velocities, those whose discharges are coincident. Another possibility is activity-dependent modification of conduction velocity. There is now ample evidence that neural activity influences myelination by stimulation of oligodendrocytes (222). Whether these modifications of conduction velocity depend on correlations between presynaptic and postsynaptic activity, which would be required to associate inputs according to the coincidence of their discharges, is unknown. The fact that learning processes are accompanied by changes in the myelination pattern of fiber tracts (223–225) suggests that myelination might indeed be controlled by associative mechanisms. Taken together, these observations suggest the possibility that neuronal networks are structured with similarly high precision in the temporal as in the spatial dimension. If this turns out to be true, conduction delays would be an important variable of computational architectures rather than an unavoidable epiphenomenon, further emphasizing the critical role of dynamics and precise control of temporal relations in information processing.

## Concluding Remarks

The rapid switching or collapse of a high-dimensional resting state toward a lower-dimensional substate bears similarities to other processes that have been proposed to exploit dynamic interactions in reciprocally coupled systems for computations. In addition to the concepts derived from reservoir computing mentioned above (117–122), these comprise theories based on attractor dynamics, aliasing processes in Little−Hopfield nets and Ising models (226), and the concept of free energy reduction (227). Last but not least, there is a puzzling analogy with the processes that make quantum computing so fast and efficient. The superposition of wave functions bears similarities to the covert superposition of priors in the correlation structure of spontaneous activity, and the simultaneous and probabilistic evaluation of nested relations resembles the virtually simultaneous and holistic interaction between network nodes that represent, in a probabilistic and graded way, the presence of particular features. It would be truly fascinating if evolution had succeeded to realize, with classical mechanisms, those functions that quantum computers are particularly good at: the parallel and therefore ultrafast evaluation of the relations between a huge number of probabilistic variables.

## Data Availability

There are no data underlying this work.

## References

[1] H. von Helmholtz, Handbuch der Physiologischen Optik (Leopold Voss Verlag, Hamburg, Leipzig, 1867).

[2] R. P. Rao, D. H. Ballard, Predictive coding in the visual cortex: A functional interpretation of some extra-classical receptive-field effects. Nat. Neurosci. 2, 79–87 (1999). 10195184 10.1038/4580

[3] E. P. Simoncelli, B. A. Olshausen, Natural image statistics and neural representation. Annu. Rev. Neurosci. 24, 1193–1216 (2001). 11520932 10.1146/annurev.neuro.24.1.1193

[4] Y. Weiss, E. P. Simoncelli, E. H. Adelson, Motion illusions as optimal percepts. Nat. Neurosci. 5, 598–604 (2002). 12021763 10.1038/nn0602-858

[5] K. Doya, K. Ishii, A. Pouget, R. P. N. Rao, Bayesian Brain: Probabilistic Approaches to Neural Coding (MIT Press, Cambridge, MA, 2011).

[6] A. M. Bastos., Canonical microcircuits for predictive coding. Neuron 76, 695–711 (2012). 23177956 10.1016/j.neuron.2012.10.038 PMC3777738

[7] R. N. Sachdev, M. R. Krause, J. A. Mazer, Surround suppression and sparse coding in visual and barrel cortices. Front. Neural Circuits 6, 43 (2012). 22783169 10.3389/fncir.2012.00043 PMC3389675

[8] D. H. Hubel, T. N. Wiesel, Receptive fields and functional architecture of monkey striate cortex. J. Physiol. 195, 215–243 (1968). 4966457 10.1113/jphysiol.1968.sp008455 PMC1557912

[9] H. B. Barlow, Single units and sensation: A neuron doctrine for perceptual psychology? Perception 1, 371–394 (1972). 4377168 10.1068/p010371

[10] D. Ferster, C. Koch, Neuronal connections underlying orientation selectivity in cat visual cortex. Trends Neurosci. 10, 487–492 (1987).

[11] C. Kayser, R. F. Salazar, P. König, Responses to natural scenes in cat V1. J. Neurophysiol. 90, 1910–1920 (2003). 12750423 10.1152/jn.00195.2003

[12] D. J. Felleman, D. C. Van Essen, Distributed hierarchical processing in the primate cerebral cortex. Cereb. Cortex 1, 1–47 (1991). 1822724 10.1093/cercor/1.1.1-a

[13] M. F. Glasser., A multi-modal parcellation of human cerebral cortex. Nature 536, 171–178 (2016). 27437579 10.1038/nature18933 PMC4990127

[14] C. G. Gross, C. E. Rocha-Miranda, D. B. Bender, Visual properties of neurons in inferotemporal cortex of the Macaque. J. Neurophysiol. 35, 96–111 (1972). 4621506 10.1152/jn.1972.35.1.96

[15] D. Y. Tsao, W. A. Freiwald, R. B. H. Tootell, M. S. Livingstone, A cortical region consisting entirely of face-selective cells. Science 311, 670–674 (2006). 16456083 10.1126/science.1119983 PMC2678572

[16] T. Hirabayashi, D. Takeuchi, K. Tamura, Y. Miyashita, Microcircuits for hierarchical elaboration of object coding across primate temporal areas. Science 341, 191–195 (2013). 23846902 10.1126/science.1236927

[17] R. Q. Quiroga, L. Reddy, G. Kreiman, C. Koch, I. Fried, Invariant visual representation by single neurons in the human brain. Nature 435, 1102–1107 (2005). 15973409 10.1038/nature03687

[18] B. A. Olshausen, D. J. Field, Emergence of simple-cell receptive field properties by learning a sparse code for natural images. Nature 381, 607–609 (1996). 8637596 10.1038/381607a0

[19] A. J. Bell, T. J. Sejnowski, The “independent components” of natural scenes are edge filters. Vision Res. 37, 3327–3338 (1997). 9425547 10.1016/s0042-6989(97)00121-1 PMC2882863

[20] J. P. Rauschecker, W. Singer, Changes in the circuitry of the kitten visual cortex are gated by postsynaptic activity. Nature 280, 58–60 (1979). 15305579 10.1038/280058a0

[21] J. P. Rauschecker, W. Singer, The effects of early visual experience on the cat’s visual cortex and their possible explanation by Hebb synapses. J. Physiol. 310, 215–239 (1981). 7230034 10.1113/jphysiol.1981.sp013545 PMC1274736

[22] C. Blakemore, G. F. Cooper, Development of the brain depends on the visual environment. Nature 228, 477–478 (1970). 5482506 10.1038/228477a0

[23] G. G. Blasdel, D. E. Mitchell, D. W. Muir, J. D. Pettigrew, A physiological and behavioural study in cats of the effect of early visual experience with contours of a single orientation. J. Physiol. 265, 615–636 (1977). 853380 10.1113/jphysiol.1977.sp011734 PMC1307838

[24] F. Tretter, M. Cynader, W. Singer, Modification of direction selectivity of neurons in the visual cortex of kittens. Brain Res. 84, 143–149 (1975). 1111822 10.1016/0006-8993(75)90808-2

[25] W. Singer, F. Tretter, Unusually large receptive fields in cats with restricted visual experience. Exp. Brain Res. 26, 171–184 (1976). 976399 10.1007/BF00238281

[26] M. Oberlaender, A. Ramirez, R. M. Bruno, Sensory experience restructures thalamocortical axons during adulthood. Neuron 74, 648–655 (2012). 22632723 10.1016/j.neuron.2012.03.022 PMC3564553

[27] C. D. Gilbert, W. Li, V. Piech, Perceptual learning and adult cortical plasticity. J. Physiol. 587, 2743–2751 (2009). 19525560 10.1113/jphysiol.2009.171488 PMC2718234

[28] A. Karni, D. Sagi, Where practice makes perfect in texture discrimination: Evidence for primary visual cortex plasticity. Proc. Natl. Acad. Sci. U.S.A. 88, 4966–4970 (1991). 2052578 10.1073/pnas.88.11.4966 PMC51788

[29] A. Schoups, R. Vogels, N. Qian, G. Orban, Practising orientation identification improves orientation coding in V1 neurons. Nature 412, 549–553 (2001). 11484056 10.1038/35087601

[30] K. Sakai, Y. Miyashita, Neural organization for the long-term memory of paired associates. Nature 354, 152–155 (1991). 1944594 10.1038/354152a0

[31] F. Rosenblatt, The perceptron: A probabilistic model for information storage and organization in the brain. Psychol. Rev. 65, 386–408 (1958). 13602029 10.1037/h0042519

[32] J. J. Hopfield, Learning algorithms and probability distributions in feed-forward and feed-back networks. Proc. Natl. Acad. Sci. U.S.A. 84, 8429–8433 (1987). 16593901 10.1073/pnas.84.23.8429 PMC299557

[33] J. J. DiCarlo, D. D. Cox, Untangling invariant object recognition. Trends Cogn. Sci. 11, 333–341 (2007). 17631409 10.1016/j.tics.2007.06.010

[34] Y. LeCun, Y. Bengio, G. Hinton, Deep learning. Nature 521, 436–444 (2015). 26017442 10.1038/nature14539

[35] D. Silver., Mastering the game of Go without human knowledge. Nature 550, 354–359 (2017). 29052630 10.1038/nature24270

[36] D. Silver., A general reinforcement learning algorithm that masters chess, shogi, and Go through self-play. Science 362, 1140–1144 (2018). 30523106 10.1126/science.aar6404

[37] W. Dabney., A distributional code for value in dopamine-based reinforcement learning. Nature 577, 671–675 (2020). 31942076 10.1038/s41586-019-1924-6 PMC7476215

[38] S. Hochreiter, J. Schmidhuber, Long short-term memory. Neural Comput. 9, 1735–1780 (1997). 9377276 10.1162/neco.1997.9.8.1735

[39] A.Banino., Vector-based navigation using grid-like representations in artificial agents. Nature557, 429–433 (2018).2974367010.1038/s41586-018-0102-6

[40] D. O. Hebb, The Organization of Behavior (John Wiley, New York, 1949). 10.1016/s0361-9230(99)00182-3 10643472

[41] N. T. Markov., A weighted and directed interareal connectivity matrix for macaque cerebral cortex. Cereb. Cortex 24, 17–36 (2014). 23010748 10.1093/cercor/bhs270 PMC3862262

[42] S. W. Oh., A mesoscale connectome of the mouse brain. Nature 508, 207–214 (2014). 24695228 10.1038/nature13186 PMC5102064

[43] S. Peron., Recurrent interactions in local cortical circuits. Nature 579, 256–259 (2020). 32132709 10.1038/s41586-020-2062-x PMC8092186

[44] W. Singer, Neuronal synchrony: A versatile code for the definition of relations? Neuron 24, 49–65, 111–125 (1999). 10677026 10.1016/s0896-6273(00)80821-1

[45] C. von der Malsburg, W. Schneider, A neural cocktail-party processor. Biol. Cybern. 54, 29–40 (1986). 3719028 10.1007/BF00337113

[46] P. M. Milner, The functional nature of neuronal oscillations. Trends Neurosci. 15, 387 (1992). 10.1016/0166-2236(92)90186-c 1279860

[47] P. M. Milner, Reply. Trends Neurosci. 15, 387–388 (1992). 10.1016/0166-2236(92)90186-c 1279860

[48] B. B. Averbeck, P. E. Latham, A. Pouget, Neural correlations, population coding and computation. Nat. Rev. Neurosci. 7, 358–366 (2006). 16760916 10.1038/nrn1888

[49] C. M. Gray, W. Singer, Stimulus-specific neuronal oscillations in orientation columns of cat visual cortex. Proc. Natl. Acad. Sci. U.S.A. 86, 1698–1702 (1989). 2922407 10.1073/pnas.86.5.1698 PMC286768

[50] C. M. Gray, P. König, A. K. Engel, W. Singer, Oscillatory responses in cat visual cortex exhibit inter-columnar synchronization which reflects global stimulus properties. Nature 338, 334–337 (1989). 2922061 10.1038/338334a0

[51] M. Abeles, Corticonics (Cambridge University Press, Cambridge, United Kingdom, 1991).

[52] R. M. Bruno, B. Sakmann, Cortex is driven by weak but synchronously active thalamocortical synapses. Science 312, 1622–1627 (2006). 16778049 10.1126/science.1124593

[53] E. Salinas, T. J. Sejnowski, Correlated neuronal activity and the flow of neural information. Nat. Rev. Neurosci. 2, 539–550 (2001). 11483997 10.1038/35086012 PMC2868968

[54] C. von der Malsburg, J. Buhmann, Sensory segmentation with coupled neural oscillators. Biol. Cybern. 67, 233–242 (1992). 1498189 10.1007/BF00204396

[55] W. Singer, C. M. Gray, Visual feature integration and the temporal correlation hypothesis. Annu. Rev. Neurosci. 18, 555–586 (1995). 7605074 10.1146/annurev.ne.18.030195.003011

[56] R. VanRullen, R. Guyonneau, S. J. Thorpe, Spike times make sense. Trends Neurosci. 28, 1–4 (2005). 15626490 10.1016/j.tins.2004.10.010

[57] M. P. Stryker, Is grandmother an oscillation? Nature 338, 297–298 (1989). 2922058 10.1038/338297a0

[58] P. Fries, A mechanism for cognitive dynamics: Neuronal communication through neuronal coherence. Trends Cogn. Sci. 9, 474–480 (2005). 16150631 10.1016/j.tics.2005.08.011

[59] P. Fries, Rhythms for cognition: Communication through coherence. Neuron 88, 220–235 (2015). 26447583 10.1016/j.neuron.2015.09.034 PMC4605134

[60] G. Buzsáki, Rhythms of the Brain (Oxford University Press, Oxford, United Kingdom, 2006).

[61] G. Buzsáki, N. Logothetis, W. Singer, Scaling brain size, keeping timing: Evolutionary preservation of brain rhythms. Neuron 80, 751–764 (2013). 24183025 10.1016/j.neuron.2013.10.002 PMC4009705

[62] W. Singer, Neuronal oscillations: Unavoidable and useful? Eur. J. Neurosci. 48, 2389–2398 (2018). 29247490 10.1111/ejn.13796

[63] J. O’Keefe, M. L. Recce, Phase relationship between hippocampal place units and the EEG theta rhythm. Hippocampus 3, 317–330 (1993). 8353611 10.1002/hipo.450030307

[64] P. H. Tiesinga, T. J. Sejnowski, Mechanisms for phase shifting in cortical networks and their role in communication through coherence. Front. Hum. Neurosci. 4, 196 (2010). 21103013 10.3389/fnhum.2010.00196 PMC2987601

[65] M. Vinck., Gamma-phase shifting in awake monkey visual cortex. J. Neurosci. 30, 1250–1257 (2010). 20107053 10.1523/JNEUROSCI.1623-09.2010 PMC6633799

[66] P. König, A. K. Engel, W. Singer, Integrator or coincidence detector? The role of the cortical neuron revisited. Trends Neurosci. 19, 130–137 (1996). 8658595 10.1016/s0166-2236(96)80019-1

[67] D. H. Ballard, J. F. Jehee, Dual roles for spike signaling in cortical neural populations. Front. Comput. Neurosci. 5, 22 (2011). 21687798 10.3389/fncom.2011.00022 PMC3108387

[68] M. N. Havenith., Synchrony makes neurons fire in sequence, and stimulus properties determine who is ahead. J. Neurosci. 31, 8570–8584 (2011). 21653861 10.1523/JNEUROSCI.2817-10.2011 PMC6623348

[69] M. S. Livingstone, Oscillatory firing and interneuronal correlations in squirrel monkey striate cortex. J. Neurophysiol. 75, 2467–2485 (1996). 8793757 10.1152/jn.1996.75.6.2467

[70] A. K. Kreiter, W. Singer, Stimulus-dependent synchronization of neuronal responses in the visual cortex of the awake macaque monkey. J. Neurosci. 16, 2381–2396 (1996). 8601818 10.1523/JNEUROSCI.16-07-02381.1996 PMC6578521

[71] M. Castelo-Branco, R. Goebel, S. Neuenschwander, W. Singer, Neural synchrony correlates with surface segregation rules. Nature 405, 685–689 (2000). 10864325 10.1038/35015079

[72] A. K. Engel, P. König, W. Singer, Direct physiological evidence for scene segmentation by temporal coding. Proc. Natl. Acad. Sci. U.S.A. 88, 9136–9140 (1991). 1924376 10.1073/pnas.88.20.9136 PMC52667

[73] P. König, T. B. Schillen, Stimulus-dependent assembly formation of oscillatory responses: I. Synchronization. Neural Comput. 3, 155–166 (1991). 31167303 10.1162/neco.1991.3.2.155

[74] T. B. Schillen, P. König, Stimulus-dependent assembly formation of oscillatory responses: II. Desynchronization. Neural Comput. 3, 167–178 (1991). 31167305 10.1162/neco.1991.3.2.167

[75] T. B. Schillen, P. König, Binding by temporal structure in multiple feature domains of an oscillatory neuronal network. Biol. Cybern. 70, 397–405 (1994). 8186300 10.1007/BF00203232

[76] W. Singer, Synchronization of cortical activity and its putative role in information processing and learning. Annu. Rev. Physiol. 55, 349–374 (1993). 8466179 10.1146/annurev.ph.55.030193.002025

[77] S. Ray, J. H. R. Maunsell, Differences in gamma frequencies across visual cortex restrict their possible use in computation. Neuron 67, 885–896 (2010). 20826318 10.1016/j.neuron.2010.08.004 PMC3001273

[78] M. N. Shadlen, J. A. Movshon, Synchrony unbound: A critical evaluation of the temporal binding hypothesis. Neuron 24, 67–77, 111–125 (1999). 10677027 10.1016/s0896-6273(00)80822-3

[79] M. Chen., Incremental integration of global contours through interplay between visual cortical areas. Neuron 82, 682–694 (2014). 24811385 10.1016/j.neuron.2014.03.023

[80] S. Ray, J. H. R. Maunsell, Do gamma oscillations play a role in cerebral cortex? Trends Cogn. Sci. 19, 78–85 (2015). 25555444 10.1016/j.tics.2014.12.002 PMC5403517

[81] E. Lowet, M. J. Roberts, P. Bonizzi, J. Karel, P. De Weerd, Quantifying neural oscillatory synchronization: A comparison between spectral coherence and phase-locking value approaches. PLoS One 11, e0146443 (2016). 26745498 10.1371/journal.pone.0146443 PMC4706353

[82] E. Lowet, M. J. Roberts, B. Gips, P. De Weerd, A. Peter, A quantitative theory of gamma synchronization in macaque V1. eLife 6, e26642 (2017). 28857743 10.7554/eLife.26642 PMC5779232

[83] M. Lundqvist., Gamma and beta bursts underlie working memory. Neuron 90, 152–164 (2016). 26996084 10.1016/j.neuron.2016.02.028 PMC5220584

[84] L. Chauvière, W. Singer, Neurofeedback training of gamma oscillations in monkey primary visual cortex. Cereb. Cortex 29, 4785–4802 (2019). 30796824 10.1093/cercor/bhz013

[85] A. Palmigiano, T. Geisel, F. Wolf, D. Battaglia, Flexible information routing by transient synchrony. Nat. Neurosci. 20, 1014–1022 (2017). 28530664 10.1038/nn.4569

[86] C. Korndörfer, E. Ullner, J. García-Ojalvo, G. Pipa, Cortical spike synchrony as a measure of input familiarity. Neural Comput. 29, 2491–2510 (2017). 28599117 10.1162/NECO_a_00987

[87] P. R. Roelfsema, V. A. F. Lamme, H. Spekreijse, Synchrony and covariation of firing rates in the primary visual cortex during contour grouping. Nat. Neurosci. 7, 982–991 (2004). 15322549 10.1038/nn1304

[88] T. Hirabayashi, Y. Miyashita, Dynamically modulated spike correlation in monkey inferior temporal cortex depending on the feature configuration within a whole object. J. Neurosci. 25, 10299–10307 (2005). 16267238 10.1523/JNEUROSCI.3036-05.2005 PMC6725794

[89] C. D. Gilbert, T. N. Wiesel, Clustered intrinsic connections in cat visual cortex. J. Neurosci. 3, 1116–1133 (1983). 6188819 10.1523/JNEUROSCI.03-05-01116.1983 PMC6564507

[90] K. S. Rockland, J. S. Lund, Intrinsic laminar lattice connections in primate visual cortex. J. Comp. Neurol. 216, 303–318 (1983). 6306066 10.1002/cne.902160307

[91] J. S. Lund, A. Angelucci, P. C. Bressloff, Anatomical substrates for functional columns in macaque monkey primary visual cortex. Cereb. Cortex 13, 15–24 (2003). 12466211 10.1093/cercor/13.1.15

[92] C. D. Gilbert, T. N. Wiesel, Columnar specificity of intrinsic horizontal and corticocortical connections in cat visual cortex. J. Neurosci. 9, 2432–2442 (1989). 2746337 10.1523/JNEUROSCI.09-07-02432.1989 PMC6569760

[93] K. E. Schmidt, R. Goebel, S. Löwel, W. Singer, The perceptual grouping criterion of colinearity is reflected by anisotropies of connections in the primary visual cortex. Eur. J. Neurosci. 9, 1083–1089 (1997). 9182961 10.1111/j.1460-9568.1997.tb01459.x

[94] K. E. Schmidt, D.-S. Kim, W. Singer, T. Bonhoeffer, S. Löwel, Functional specificity of long-range intrinsic and interhemispheric connections in the visual cortex of strabismic cats. J. Neurosci. 17, 5480–5492 (1997). 9204930 10.1523/JNEUROSCI.17-14-05480.1997 PMC6793806

[95] W. H. Bosking, Y. Zhang, B. Schofield, D. Fitzpatrick, Orientation selectivity and the arrangement of horizontal connections in tree shrew striate cortex. J. Neurosci. 17, 2112–2127 (1997). 9045738 10.1523/JNEUROSCI.17-06-02112.1997 PMC6793759

[96] M. Okun., Diverse coupling of neurons to populations in sensory cortex. Nature 521, 511–515 (2015). 25849776 10.1038/nature14273 PMC4449271

[97] S. A. Conde-Ocazionez., Callosal influence on visual receptive fields has an ocular, an orientation- and direction bias. Front. Syst. Neurosci. 12, 11 (2018). 29713267 10.3389/fnsys.2018.00011 PMC5911488

[98] J. C. Houzel, C. Milleret, G. Innocenti, Morphology of callosal axons interconnecting areas 17 and 18 of the cat. Eur. J. Neurosci. 6, 898–917 (1994). 7952278 10.1111/j.1460-9568.1994.tb00585.x

[99] A. K. Engel, P. König, A. K. Kreiter, W. Singer, Interhemispheric synchronization of oscillatory neuronal responses in cat visual cortex. Science 252, 1177–1179 (1991). 2031188 10.1126/science.252.5009.1177

[100] R. Vicente, L. L. Gollo, C. R. Mirasso, I. Fischer, G. Pipa, Dynamical relaying can yield zero time lag neuronal synchrony despite long conduction delays. Proc. Natl. Acad. Sci. U.S.A. 105, 17157–17162 (2008). 18957544 10.1073/pnas.0809353105 PMC2575223

[101] P. Bush, T. Sejnowski, Inhibition synchronizes sparsely connected cortical neurons within and between columns in realistic network models. J. Comput. Neurosci. 3, 91–110 (1996). 8840227 10.1007/BF00160806

[102] M. P. Jadi, T. J. Sejnowski, Cortical oscillations arise from contextual interactions that regulate sparse coding. Proc. Natl. Acad. Sci. U.S.A. 111, 6780–6785 (2014). 24742427 10.1073/pnas.1405300111 PMC4020078

[103] A. Knoblauch, F. T. Sommer, Synaptic plasticity, conduction delays, and inter-areal phase relations of spike activity in a model of reciprocally connected areas. Neurocomputing 52, 301–306 (2003).

[104] A. Knoblauch, F. T. Sommer, Spike-timing-dependent synaptic plasticity can form “zero lag links” for cortical oscillations. Neurocomputing 58, 185–190 (2004).

[105] M. Massimini., Breakdown of cortical effective connectivity during sleep. Science 309, 2228–2232 (2005). 16195466 10.1126/science.1117256

[106] L. Muller, F. Chavane, J. Reynolds, T. J. Sejnowski, Cortical travelling waves: Mechanisms and computational principles. Nat. Rev. Neurosci. 19, 255–268 (2018). 29563572 10.1038/nrn.2018.20 PMC5933075

[107] D. Plenz, T. C. Thiagarajan, The organizing principles of neuronal avalanches: Cell assemblies in the cortex? Trends Neurosci. 30, 101–110 (2007). 17275102 10.1016/j.tins.2007.01.005

[108] G. Hahn., Neuronal avalanches in spontaneous activity in vivo. J. Neurophysiol. 104, 3312–3322 (2010). 20631221 10.1152/jn.00953.2009 PMC3007625

[109] G. B. Ermentrout, D. Kleinfeld, Traveling electrical waves in cortex: Insights from phase dynamics and speculation on a computational role. Neuron 29, 33–44 (2001). 11182079 10.1016/s0896-6273(01)00178-7

[110] B. Lima, W. Singer, S. Neuenschwander, Gamma responses correlate with temporal expectation in monkey primary visual cortex. J. Neurosci. 31, 15919–15931 (2011). 22049435 10.1523/JNEUROSCI.0957-11.2011 PMC6623032

[111] A. Peter., Surface color and predictability determine contextual modulation of V1 firing and gamma oscillations. eLife 8, e42101 (2019). 30714900 10.7554/eLife.42101 PMC6391066

[112] J. Biederlack., Brightness induction: Rate enhancement and neuronal synchronization as complementary codes. Neuron 52, 1073–1083 (2006). 17178409 10.1016/j.neuron.2006.11.012

[113] X. Jia, D. Xing, A. Kohn, No consistent relationship between gamma power and peak frequency in macaque primary visual cortex. J. Neurosci. 33, 17–25 (2013). 23283318 10.1523/JNEUROSCI.1687-12.2013 PMC3560843

[114] M. A. Gieselmann, A. Thiele, Comparison of spatial integration and surround suppression characteristics in spiking activity and the local field potential in macaque V1. Eur. J. Neurosci. 28, 447–459 (2008). 18702717 10.1111/j.1460-9568.2008.06358.x

[115] C. Uran., Predictability in natural images determines V1 firing rates and synchronization: A deep neural network approach. bioRxiv [Preprint] (2020). 10.1101/2020.08.10.242958 (Accessed 22 July 2021).

[116] M. Vinck, C. A. Bosman, More gamma more predictions: Gamma-synchronization as a key mechanism for efficient integration of classical receptive field inputs with surround predictions. Front. Syst. Neurosci. 10, 35 (2016). 27199684 10.3389/fnsys.2016.00035 PMC4842768

[117] M. Lukoševičius, H. Jaeger, Reservoir computing approaches to recurrent neural network training. Comput. Sci. Rev. 3, 127–149 (2009).

[118] D. V. Buonomano, W. Maass, State-dependent computations: Spatiotemporal processing in cortical networks. Nat. Rev. Neurosci. 10, 113–125 (2009). 19145235 10.1038/nrn2558

[119] O. D’Huys, I. Fischer, J. Danckaert, R. Vicente, Spectral and correlation properties of rings of delay-coupled elements: Comparing linear and nonlinear systems. Phys. Rev. E Stat. Nonlin. Soft Matter Phys. 85, 056209 (2012). 23004845 10.1103/PhysRevE.85.056209

[120] M. C. Soriano, J. Garcia-Ojalvo, C. R. Mirasso, I. Fischer, Complex photonics: Dynamics and applications of delay-coupled semiconductors lasers. Rev. Mod. Phys. 85, 421–470 (2013).

[121] A. Lazar, G. Pipa, J. Triesch, SORN: A self-organizing recurrent neural network. Front. Comput. Neurosci. 3, 23 (2009). 19893759 10.3389/neuro.10.023.2009 PMC2773171

[122] M. Romera., Vowel recognition with four coupled spin-torque nano-oscillators. Nature 563, 230–234 (2018). 30374193 10.1038/s41586-018-0632-y

[123] G. Bellec., Biologically inspired alternatives to backpropagation through time for learning in recurrent neural nets. arXiv [Preprint] (2019). https://arxiv.org/abs/1901.09049 (Accessed 22 July 2021).

[124] S. Habenschuss, Z. Jonke, W. Maass, Stochastic computations in cortical microcircuit models. PLoS Comput. Biol. 9, e1003311 (2013). 24244126 10.1371/journal.pcbi.1003311 PMC3828141

[125] L. Buesing, J. Bill, B. Nessler, W. Maass, Neural dynamics as sampling: A model for stochastic computation in recurrent networks of spiking neurons. PLoS Comput. Biol. 7, e1002211 (2011). 22096452 10.1371/journal.pcbi.1002211 PMC3207943

[126] D. Nikolić, S. Häusler, W. Singer, W. Maass, Distributed fading memory for stimulus properties in the primary visual cortex. PLoS Biol. 7, e1000260 (2009). 20027205 10.1371/journal.pbio.1000260 PMC2785877

[127] W. Singer, Cortical dynamics revisited. Trends Cogn. Sci. 17, 616–626 (2013). 24139950 10.1016/j.tics.2013.09.006

[128] W. Singer, A. Lazar, Does the cerebral cortex exploit high-dimensional, non-linear dynamics for information processing? Front. Comput. Neurosci. 10, 99 (2016). 27713697 10.3389/fncom.2016.00099 PMC5031693

[129] M. Rigotti., The importance of mixed selectivity in complex cognitive tasks. Nature 497, 585–590 (2013). 23685452 10.1038/nature12160 PMC4412347

[130] D. A. McCormick, C. M. Gray, Z. Wang, Chattering cells: A new physiological subtype which may contribute to 20–60 Hz oscillations in cat visual cortex. Soc. Neurosci. Abstr. 19, 869 (1993).

[131] I. Onorato., A distinct class of bursting neurons with strong gamma synchronization and stimulus selectivity in monkey V1. Neuron 105, 180–197.e5 (2020). 31732258 10.1016/j.neuron.2019.09.039

[132] N. Kopell, G. B. Ermentrout, M. A. Whittington, R. D. Traub, Gamma rhythms and beta rhythms have different synchronization properties. Proc. Natl. Acad. Sci. U.S.A. 97, 1867–1872 (2000). 10677548 10.1073/pnas.97.4.1867 PMC26528

[133] S. Fusi, E. K. Miller, M. Rigotti, Why neurons mix: High dimensionality for higher cognition. Curr. Opin. Neurobiol. 37, 66–74 (2016). 26851755 10.1016/j.conb.2016.01.010

[134] O. Yizhar., Neocortical excitation/inhibition balance in information processing and social dysfunction. Nature 477, 171–178 (2011). 21796121 10.1038/nature10360 PMC4155501

[135] G. G. Turrigiano, S. B. Nelson, Homeostatic plasticity in the developing nervous system. Nat. Rev. Neurosci. 5, 97–107 (2004). 14735113 10.1038/nrn1327

[136] T. V. P. Bliss, T. Lomo, Long-lasting potentiation of synaptic transmission in the dentate area of the anaesthetized rabbit following stimulation of the perforant path. J. Physiol. 232, 331–356 (1973). 4727084 10.1113/jphysiol.1973.sp010273 PMC1350458

[137] A. Kohn, J. A. Movshon, Neuronal adaptation to visual motion in area MT of the macaque. Neuron 39, 681–691 (2003). 12925281 10.1016/s0896-6273(03)00438-0

[138] R. C. Muresan, C. Savin, Resonance or integration? Self-sustained dynamics and excitability of neural microcircuits. J. Neurophysiol. 97, 1911–1930 (2007). 17135469 10.1152/jn.01043.2006

[139] G. Aston-Jones, J. D. Cohen, An integrative theory of locus coeruleus-norepinephrine function: Adaptive gain and optimal performance. Annu. Rev. Neurosci. 28, 403–450 (2005). 16022602 10.1146/annurev.neuro.28.061604.135709

[140] K. D. Harris, A. Thiele, Cortical state and attention. Nat. Rev. Neurosci. 12, 509–523 (2011). 21829219 10.1038/nrn3084 PMC3324821

[141] G. Spyropoulos., Spontaneous variability in gamma dynamics described by a linear harmonic oscillator driven by noise. bioRxiv [Preprint] (2020). 10.1101/793729 (Accessed 22 July 2021). PMC9018758 35440540

[142] J. Wilting, V. Priesemann, 25 years of criticality in neuroscience - Established results, open controversies, novel concepts. Curr. Opin. Neurobiol. 58, 105–111 (2019). 31546053 10.1016/j.conb.2019.08.002

[143] N. Bertschinger, T. Natschläger, Real-time computation at the edge of chaos in recurrent neural networks. Neural Comput. 16, 1413–1436 (2004). 15165396 10.1162/089976604323057443

[144] J. M. Beggs, N. Timme, Being critical of criticality in the brain. Front. Physiol. 3, 163 (2012). 22701101 10.3389/fphys.2012.00163 PMC3369250

[145] M. W. Spratling, A review of predictive coding algorithms. Brain Cogn. 112, 92–97 (2017). 26809759 10.1016/j.bandc.2015.11.003

[146] J. Bruineberg, E. Rietveld, T. Parr, L. van Maanen, K. J. Friston, Free-energy minimization in joint agent-environment systems: A niche construction perspective. J. Theor. Biol. 455, 161–178 (2018). 30012517 10.1016/j.jtbi.2018.07.002 PMC6117456

[147] S. Musall, M. T. Kaufman, A. L. Juavinett, S. Gluf, A. K. Churchland, Single-trial neural dynamics are dominated by richly varied movements. Nat. Neurosci. 22, 1677–1686 (2019). 31551604 10.1038/s41593-019-0502-4 PMC6768091

[148] C. Stringer., Spontaneous behaviors drive multidimensional, brainwide activity. Science 364, 255–266 (2019). 31000656 10.1126/science.aav7893 PMC6525101

[149] R. G. Bettinardi., How structure sculpts function: Unveiling the contribution of anatomical connectivity to the brain’s spontaneous correlation structure. Chaos 27, 047409 (2017). 28456160 10.1063/1.4980099

[150] T. Kenet, D. Bibitchkov, M. Tsodyks, A. Grinvald, A. Arieli, Spontaneously emerging cortical representations of visual attributes. Nature 425, 954–956 (2003). 14586468 10.1038/nature02078

[151] D. Y. Ts’o, C. D. Gilbert, T. N. Wiesel, Relationships between horizontal interactions and functional architecture in cat striate cortex as revealed by cross-correlation analysis. J. Neurosci. 6, 1160–1170 (1986). 3701413 10.1523/JNEUROSCI.06-04-01160.1986 PMC6568437

[152] P. Berkes, G. Orbán, M. Lengyel, J. Fiser, Spontaneous cortical activity reveals hallmarks of an optimal internal model of the environment. Science 331, 83–87 (2011). 21212356 10.1126/science.1195870 PMC3065813

[153] A. Angelucci, J. B. Levitt, J. S. Lund, Anatomical origins of the classical receptive field and modulatory surround field of single neurons in macaque visual cortical area V1. Prog. Brain Res. 136, 373–388 (2002). 12143395 10.1016/s0079-6123(02)36031-x

[154] L. Nurminen, S. Merlin, M. Bijanzadeh, F. Federer, A. Angelucci, Top-down feedback controls spatial summation and response amplitude in primate visual cortex. Nat. Commun. 9, 2281 (2018). 29892057 10.1038/s41467-018-04500-5 PMC5995810

[155] M. Bányai., Stimulus complexity shapes response correlations in primary visual cortex. Proc. Natl. Acad. Sci. U.S.A. 116, 2723–2732 (2019). 30692266 10.1073/pnas.1816766116 PMC6377442

[156] D. H. Hubel, T. N. Wiesel, Receptive fields of cells in striate cortex of very young, visually inexperienced kittens. J. Neurophysiol. 26, 994–1002 (1963). 14084171 10.1152/jn.1963.26.6.994

[157] W. Singer, Development and plasticity of cortical processing architectures. Science 270, 758–764 (1995). 7481762 10.1126/science.270.5237.758

[158] W. Singer, “The role of oscillations and synchrony in the development of the nervous system” in Emergent Brain Dynamics. Prebirth to Adolescence. Strüngmann Forum Reports, A. A. Benasich, U. Ribary, Eds. (MIT Press, Cambridge, MA, 2018), pp. 15–32.

[159] S. Löwel, W. Singer, Selection of intrinsic horizontal connections in the visual cortex by correlated neuronal activity. Science 255, 209–212 (1992). 1372754 10.1126/science.1372754

[160] P. Buisseret, W. Singer, Proprioceptive signals from extraocular muscles gate experience dependent modifications of receptive fields in the kitten visual cortex. Exp. Brain Res. 51, 443–450 (1983).

[161] M. F. Bear, W. Singer, Modulation of visual cortical plasticity by acetylcholine and noradrenaline. Nature 320, 172–176 (1986). 3005879 10.1038/320172a0

[162] W. Singer, J. P. Rauschecker, Central core control of developmental plasticity in the kitten visual cortex: II. Electrical activation of mesencephalic and diencephalic projections. Exp. Brain Res. 47, 223–233 (1982). 7117447 10.1007/BF00239381

[163] P. Buisseret, E. Gary-Bobo, M. Imbert, Ocular motility and recovery of orientational properties of visual cortical neurones in dark-reared kittens. Nature 272, 816–817 (1978). 643071 10.1038/272816a0

[164] W. Singer, F. Tretter, U. Yinon, Central gating of developmental plasticity in kitten visual cortex. J. Physiol. 324, 221–237 (1982). 7097598 10.1113/jphysiol.1982.sp014108 PMC1250701

[165] W. Singer, U. Yinon, F. Tretter, Inverted monocular vision prevents ocular dominance shift in kittens and impairs the functional state of visual cortex in adult cats. Brain Res. 164, 294–299 (1979). 427562 10.1016/0006-8993(79)90024-6

[166] R. A. W. Galuske, M. H. J. Munk, W. Singer, Relation between gamma oscillations and neuronal plasticity in the visual cortex. Proc. Natl. Acad. Sci. U.S.A. 116, 23317–23325 (2019). 31659040 10.1073/pnas.1901277116 PMC6859324

[167] V. V. Moca, A. Nagy-Dabacan, H. Barzan, R. C. Muresan, Superlets: Time-frequency super-resolution using wavelet sets. bioRxiv [Preprint] (2019). 10.1101/583732 (Accessed 22 July 2021).

[168] V. Priesemann., Spike avalanches *in vivo* suggest a driven, slightly subcritical brain state. Front. Syst. Neurosci. 8, 108 (2014). 25009473 10.3389/fnsys.2014.00108 PMC4068003

[169] P. Fries, S. Neuenschwander, A. K. Engel, R. Goebel, W. Singer, Rapid feature selective neuronal synchronization through correlated latency shifting. Nat. Neurosci. 4, 194–200 (2001). 11175881 10.1038/84032

[170] G. B. Smith, B. Hein, D. E. Whitney, D. Fitzpatrick, M. Kaschube, Distributed network interactions and their emergence in developing neocortex. Nat. Neurosci. 21, 1600–1608 (2018). 30349107 10.1038/s41593-018-0247-5 PMC6371984

[171] C. M. Lewis, A. Baldassarre, G. Committeri, G. L. Romani, M. Corbetta, Learning sculpts the spontaneous activity of the resting human brain. Proc. Natl. Acad. Sci. U.S.A. 106, 17558–17563 (2009). 19805061 10.1073/pnas.0902455106 PMC2762683

[172] B. Kundu, D. W. Sutterer, S. M. Emrich, B. R. Postle, Strengthened effective connectivity underlies transfer of working memory training to tests of short-term memory and attention. J. Neurosci. 33, 8705–8715 (2013). 23678114 10.1523/JNEUROSCI.5565-12.2013 PMC3758887

[173] M. M. Churchland., Stimulus onset quenches neural variability: A widespread cortical phenomenon. Nat. Neurosci. 13, 369–378 (2010). 20173745 10.1038/nn.2501 PMC2828350

[174] B. Lima, W. Singer, N.-H. Chen, S. Neuenschwander, Synchronization dynamics in response to plaid stimuli in monkey V1. Cereb. Cortex 20, 1556–1573 (2010). 19812238 10.1093/cercor/bhp218 PMC2882822

[175] A. Lazar, C. Lewis, P. Fries, W. Singer, D. Nikolić, Visual exposure optimizes stimulus encoding in primary visual cortex. bioRxiv [Preprint] (2018). 10.1101/502328 (Accessed 27 July 2021). PMC8639370 34663727

[176] Yang, Y., “Dynamics of neural population activity in monkey visual cortex: Natural stimuli, temporal sequences and working memory,” PhD thesis, Johann Wolfgang Goethe University, Frankfurt, Germany (2021).

[177] F. Han, N. Caporale, Y. Dan, Reverberation of recent visual experience in spontaneous cortical waves. Neuron 60, 321–327 (2008). 18957223 10.1016/j.neuron.2008.08.026 PMC3576032

[178] C. Hartmann, A. Lazar, B. Nessler, J. Triesch, Where’s the noise? Key features of spontaneous activity and neural variability arise through learning in a deterministic network. PLoS Comput. Biol. 11, e1004640 (2015). 26714277 10.1371/journal.pcbi.1004640 PMC4694925

[179] D. E. Feldman, The spike-timing dependence of plasticity. Neuron 75, 556–571 (2012). 22920249 10.1016/j.neuron.2012.08.001 PMC3431193

[180] M. R. Cohen, J. H. Maunsell, Attention improves performance primarily by reducing interneuronal correlations. Nat. Neurosci. 12, 1594–1600 (2009). 19915566 10.1038/nn.2439 PMC2820564

[181] J. F. Mitchell, K. A. Sundberg, J. H. Reynolds, Spatial attention decorrelates intrinsic activity fluctuations in macaque area V4. Neuron 63, 879–888 (2009). 19778515 10.1016/j.neuron.2009.09.013 PMC2765230

[182] M. Chalk., Attention reduces stimulus-driven gamma frequency oscillations and spike field coherence in V1. Neuron 66, 114–125 (2010). 20399733 10.1016/j.neuron.2010.03.013 PMC2923752

[183] M. Vinck, T. Womelsdorf, E. A. Buffalo, R. Desimone, P. Fries, Attentional modulation of cell-class-specific gamma-band synchronization in awake monkey area v4. Neuron 80, 1077–1089 (2013). 24267656 10.1016/j.neuron.2013.08.019 PMC3840396

[184] P. Fries, J. H. Reynolds, A. E. Rorie, R. Desimone, Modulation of oscillatory neuronal synchronization by selective visual attention. Science 291, 1560–1563 (2001). 11222864 10.1126/science.1055465

[185] G. G. Gregoriou, S. J. Gotts, H. Zhou, R. Desimone, High-frequency, long-range coupling between prefrontal and visual cortex during attention. Science 324, 1207–1210 (2009). 19478185 10.1126/science.1171402 PMC2849291

[186] C. A. Bosman., Attentional stimulus selection through selective synchronization between monkey visual areas. Neuron 75, 875–888 (2012). 22958827 10.1016/j.neuron.2012.06.037 PMC3457649

[187] I. Grothe, S. D. Neitzel, S. Mandon, A. K. Kreiter, Switching neuronal inputs by differential modulations of gamma-band phase-coherence. J. Neurosci. 32, 16172–16180 (2012). 23152601 10.1523/JNEUROSCI.0890-12.2012 PMC6794021

[188] E. A. Buffalo, P. Fries, R. Landman, T. J. Buschman, R. Desimone, Laminar differences in gamma and alpha coherence in the ventral stream. Proc. Natl. Acad. Sci. U.S.A. 108, 11262–11267 (2011). 21690410 10.1073/pnas.1011284108 PMC3131344

[189] M. Schneider, B. Dann, S. Sheshadri, H. Scherberger, M. Vinck, A general theory of coherence between brain areas. bioRxiv [Preprint] (2020). 10.1101/2020.06.17.156190 (Accessed 22 July 2021).

[190] W. Köhler, Gestalt Psychology (Bells and Sons, London, United Kingdom, 1930).

[191] R. A. W. Galuske, W. Schlote, H. Bratzke, W. Singer, Interhemispheric asymmetries of the modular structure in human temporal cortex. Science 289, 1946–1949 (2000). 10988077 10.1126/science.289.5486.1946

[192] K. Kar, J. Kubilius, K. Schmidt, E. B. Issa, J. J. DiCarlo, Evidence that recurrent circuits are critical to the ventral stream’s execution of core object recognition behavior. bioRxiv [Preprint] (2018). 10.1101/354753 (Accessed 22 July 2021). PMC8785116 31036945

[193] H. Sakaguchi, S. Shinomoto, Y. Kuramoto, Local and global self-entrainment in oscillator lattices. Prog. Theor. Phys. 77, 1005–1011 (1987).

[194] S. Coombes, P. C. Bressloff, Mode locking and Arnold tongues in integrate-and-fire neural oscillators. Phys. Rev. E Stat. Phys. Plasmas Fluids Relat. Interdiscip. Topics 60, 2086–2096 (1999). 11970001 10.1103/physreve.60.2086

[195] C. M. Gray, D. A. McCormick, Chattering cells: Superficial pyramidal neurons contributing to the generation of synchronous oscillations in the visual cortex. Science 274, 109–113 (1996). 8810245 10.1126/science.274.5284.109

[196] C. Börgers, N. Kopell, Synchronization in networks of excitatory and inhibitory neurons with sparse, random connectivity. Neural Comput. 15, 509–538 (2003). 12620157 10.1162/089976603321192059

[197] M. A. Whittington, R. D. Traub, J. G. R. Jefferys, Synchronized oscillations in interneuron networks driven by metabotropic glutamate receptor activation. Nature 373, 612–615 (1995). 7854418 10.1038/373612a0

[198] M. A. Whittington, R. D. Traub, N. Kopell, B. Ermentrout, E. H. Buhl, Inhibition-based rhythms: Experimental and mathematical observations on network dynamics. Int. J. Psychophysiol. 38, 315–336 (2000). 11102670 10.1016/s0167-8760(00)00173-2

[199] M. A. Whittington, M. O. Cunningham, F. E. LeBeau, C. Racca, R. D. Traub, Multiple origins of the cortical γ rhythm. Dev. Neurobiol. 71, 92–106 (2011). 21154913 10.1002/dneu.20814

[200] A. Hasenstaub., Inhibitory postsynaptic potentials carry synchronized frequency information in active cortical networks. Neuron 47, 423–435 (2005). 16055065 10.1016/j.neuron.2005.06.016

[201] M. Bartos, I. Vida, P. Jonas, Synaptic mechanisms of synchronized gamma oscillations in inhibitory interneuron networks. Nat. Rev. Neurosci. 8, 45–56 (2007). 17180162 10.1038/nrn2044

[202] J. A. Cardin., Driving fast-spiking cells induces gamma rhythm and controls sensory responses. Nature 459, 663–667 (2009). 19396156 10.1038/nature08002 PMC3655711

[203] P. Tiesinga, T. J. Sejnowski, Cortical enlightenment: Are attentional gamma oscillations driven by ING or PING? Neuron 63, 727–732 (2009). 19778503 10.1016/j.neuron.2009.09.009 PMC2778762

[204] G. Buzsáki, X.-J. Wang, Mechanisms of gamma oscillations. Annu. Rev. Neurosci. 35, 203–225 (2012). 22443509 10.1146/annurev-neuro-062111-150444 PMC4049541

[205] V. V. Moca, D. Nikolic, W. Singer, R. C. Mureşan, Membrane resonance enables stable and robust gamma oscillations. Cereb. Cortex 24, 119–142 (2014). 23042733 10.1093/cercor/bhs293 PMC3862267

[206] D. B. Salkoff, E. Zagha, Ö. Yüzgeç, D. A. McCormick, Synaptic mechanisms of tight spike synchrony at gamma frequency in cerebral cortex. J. Neurosci. 35, 10236–10251 (2015). 26180200 10.1523/JNEUROSCI.0828-15.2015 PMC4502264

[207] S. Grillner, P. Wallén, L. Brodin, A. Lansner, Neuronal network generating locomotor behavior in lamprey: Circuitry, transmitters, membrane properties, and simulation. Annu. Rev. Neurosci. 14, 169–199 (1991). 1674412 10.1146/annurev.ne.14.030191.001125

[208] E. Marder, D. Bucher, Central pattern generators and the control of rhythmic movements. Curr. Biol. 11, R986–R996 (2001). 11728329 10.1016/s0960-9822(01)00581-4

[209] A. López-Cruz., Parallel multimodal circuits control an innate foraging behavior. Neuron 102, 407–419.e8 (2019). 30824353 10.1016/j.neuron.2019.01.053 PMC9161785

[210] Z. Wang, H. Fan, F. Han, A new regime for highly robust gamma oscillation with co-exist of accurate and weak synchronization in excitatory-inhibitory networks. Cogn. Neurodyn. 8, 335–344 (2014). 25009675 10.1007/s11571-014-9290-4 PMC4079905

[211] X. Zhang, S. Hallerberg, M. Matthiae, D. Witthaut, M. Timme, Fluctuation-induced distributed resonances in oscillatory networks. Sci. Adv. 5, eaav1027 (2019). 31392264 10.1126/sciadv.aav1027 PMC6669019

[212] E. W. Schomburg., Theta phase segregation of input-specific gamma patterns in entorhinal-hippocampal networks. Neuron 84, 470–485 (2014). 25263753 10.1016/j.neuron.2014.08.051 PMC4253689

[213] Z. W. Davis, L. Muller, J. Martinez-Trujillo, T. Sejnowski, J. H. Reynolds, Spontaneous travelling cortical waves gate perception in behaving primates. Nature 587, 432–436 (2020). 33029013 10.1038/s41586-020-2802-y PMC7677221

[214] M. H. J. Munk, P. R. Roelfsema, P. König, A. K. Engel, W. Singer, Role of reticular activation in the modulation of intracortical synchronization. Science 272, 271–274 (1996). 8602512 10.1126/science.272.5259.271

[215] R. Rodriguez, U. Kallenbach, W. Singer, M. H. J. Munk, Effects of cholinergic stimulation on gamma oscillations during visual responses in cat visual cortex. Eur. J. Neurosci. 12 (suppl. 11), 490 (2000).

[216] F. Borchard, W. Singer, M. H. J. Munk, Effects of electrical stimulation of the substantia innominata on neural activity in cat visual cortex. Eur. J. Neurosci. 12 (suppl. 11), 127 (2000).

[217] M. Steriade, R. C. Dossi, D. Paré, G. Oakson, Fast oscillations (20–40 Hz) in thalamocortical systems and their potentiation by mesopontine cholinergic nuclei in the cat. Proc. Natl. Acad. Sci. U.S.A. 88, 4396–4400 (1991). 2034679 10.1073/pnas.88.10.4396 PMC51666

[218] A. Roxin, N. Brunel, D. Hansel, Role of delays in shaping spatiotemporal dynamics of neuronal activity in large networks. Phys. Rev. Lett. 94, 238103 (2005). 16090506 10.1103/PhysRevLett.94.238103

[219] G. M. Innocenti, P. Lehmann, J. C. Houzel, Computational structure of visual callosal axons. Eur. J. Neurosci. 6, 918–935 (1994). 7952279 10.1111/j.1460-9568.1994.tb00586.x

[220] M. Brecht, W. Singer, A. K. Engel, Correlation analysis of corticotectal interactions in the cat visual system. J. Neurophysiol. 79, 2394–2407 (1998). 9582215 10.1152/jn.1998.79.5.2394

[221] J. Krüger, M. Mayer, Two types of neuronal synchrony in monkey striate cortex. Biol. Cybern. 64, 135–140 (1990). 2291901 10.1007/BF02331342

[222] R. D. Fields, A new mechanism of nervous system plasticity: Activity-dependent myelination. Nat. Rev. Neurosci. 16, 756–767 (2015). 26585800 10.1038/nrn4023 PMC6310485

[223] S. Dehaene., How learning to read changes the cortical networks for vision and language. Science 330, 1359–1364 (2010). 21071632 10.1126/science.1194140

[224] R. D. Fields, O. Bukalo, Myelin makes memories. Nat. Neurosci. 23, 469–470 (2020). 32094969 10.1038/s41593-020-0606-x PMC8240098

[225] C. Sampaio-Baptista, H. Johansen-Berg, White matter plasticity in the adult brain. Neuron 96, 1239–1251 (2017). 29268094 10.1016/j.neuron.2017.11.026 PMC5766826

[226] J. J. Hopfield, Neural networks and physical systems with emergent collective computational abilities. Proc. Natl. Acad. Sci. U.S.A. 79, 2554–2558 (1982). 6953413 10.1073/pnas.79.8.2554 PMC346238

[227] K. Friston, The free-energy principle: A unified brain theory? Nat. Rev. Neurosci. 11, 127–138 (2010). 20068583 10.1038/nrn2787

[228] R. Azouz, C. M. Gray, Adaptive coincidence detection and dynamic gain control in visual cortical neurons *in vivo*. Neuron 37, 513–523 (2003). 12575957 10.1016/s0896-6273(02)01186-8

